# Molecular Simulations of Thermal Transport across Iron Oxide–Hydrocarbon Interfaces

**DOI:** 10.1021/acsami.4c09434

**Published:** 2024-10-15

**Authors:** Fionn Carman, James P. Ewen, Fernando Bresme, Billy Wu, Daniele Dini

**Affiliations:** † Department of Mechanical Engineering, Imperial College London, London SW7 2AZ, U.K.; ‡ Department of Chemistry, Molecular Sciences Research Hub, Imperial College London, London W12 0BZ, U.K.; ¶ Dyson School of Design Engineering, Imperial College London, London SW7 2AZ, U.K.

**Keywords:** thermal management, interfacial thermal resistance, interfacial thermal conductance, thermal boundary resistance, wettability, work of adhesion, nonequilibrium molecular dynamics

## Abstract

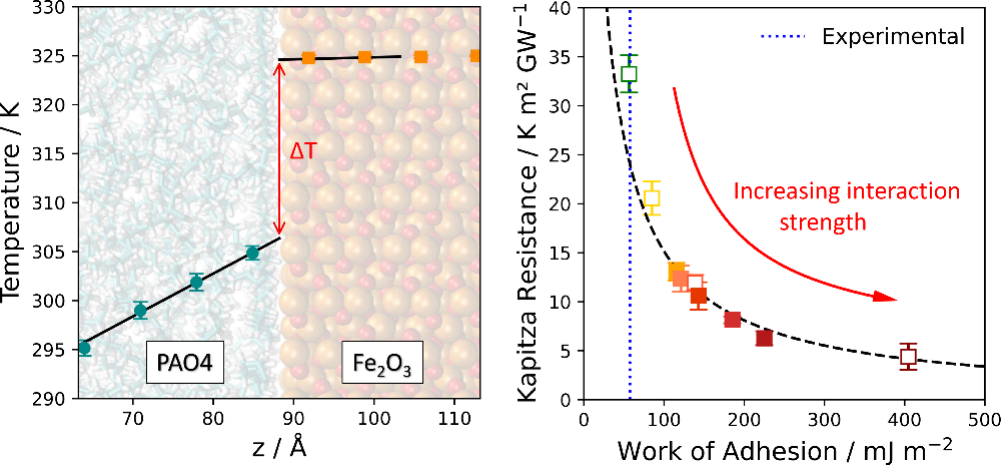

The rational design of dielectric fluids for immersion cooling of batteries requires a molecular-level understanding of the heat flow across the battery casing/dielectric fluid interface. Here, we use nonequilibrium molecular dynamics (NEMD) simulations to quantify the interfacial thermal resistance (ITR) between hematite and poly-α-olefin (PAO), which are representative of the outer surface of the steel battery casing and a synthetic hydrocarbon dielectric fluid, respectively. After identifying the most suitable force fields to model the thermal properties of the individual components, we then compared different solid–liquid interaction potentials for the calculation of the ITR. These potentials resulted in a wide range of ITR values (4–21 K m^2^ GW^–1^), with stronger solid–liquid interactions leading to lower ITR. The increase in ITR is correlated with an increase in density of the fluid layer closest to the surface. Since the ITR has not been experimentally measured for the hematite/PAO interface, we validate the solid–liquid interaction potential using the work of adhesion calculated using the dry-surface method. The work of adhesion calculations from the simulations were compared to those derived from experimental contact angle measurements for PAO on steel. We find that all of the solid–liquid potentials overestimate the experimental work of adhesion. The experiments and simulations can only be reconciled by further reducing the strength of the interfacial interactions. This suggests some screening of the solid–liquid interactions, which may be due to the presence of an interfacial water layer between PAO and steel in the contact angle experiments. Using the solid–liquid interaction potential that reproduces the experimental work of adhesion, we obtain a higher ITR (33 K m^2^ GW^–1^), suggesting inefficient thermal transport. The results of this study demonstrate the potential for NEMD simulations to improve understanding of the nanoscale thermal transport across industrially important interfaces. This study represents an important step toward the rational design of more effective fluids for immersion cooling systems for electric vehicles and other applications where thermal management is of high importance.

## Introduction

The interfacial thermal resistance (ITR), which is sometimes also referred to as the thermal boundary resistance,^1^ is a measure of the resistance to heat flow between two dissimilar materials. It is a crucial factor in understanding heat transfer across material interfaces.^2^ The ITR results in a temperature discontinuity where the two materials meet, arising from the contrasting electronic and vibrational properties of the two materials.^2^ The ITR, or its inverse, interfacial thermal conductance (ITC), is given by the equation:
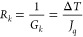
1where *R*_*k*_ and *G*_*k*_ represent the ITR and ITC respectively, *ΔT* is the temperature discontinuity at the material interface and *J*_*q*_ is the heat flux normal to the plane of the interface.

Experimental methods to study ITR, such as time-domain thermoreflectance (TDTR) and frequency-domain thermoreflectance (FDTR),^3^ are time-consuming and expensive.^2^ As a result, nonequilibrium molecular dynamics (NEMD) simulations have emerged as a popular approach to determining the ITR.^4^ NEMD simulations have been used to investigate the ITR at a wide range of solid–liquid interfaces, including: graphene/water,^5^ graphene/alkane,^6^ graphene/ionic liquid,^7^ gold/water,^8^ gold/surfactant/water,^9^ gold/surfactant/alkane,^10^ gold/surfactant/amine,^11^ quartz/water,^12^ quartz/alkane,^13^ alumina/water,^14^ silicon/water,^15^ silica/water,^16^ and silicon carbide/water.^17^

The iron oxide/alkane interface is of particular relevance to immersion cooling systems, such as those used for the thermal management of batteries for electric vehicles.^18^ Iron oxide is representative of the oxidized outer layer of steel battery casings^19^ in contact with a hydrocarbon-based coolant.^18^ Heat generated within the cell must be transferred through several interfaces to the outer case and then dissipated into the cooling fluid. To improve cooling efficiency, it is important to understand and characterize the interfacial heat transport across the many internal and external interfaces. Studies have shown that the cathode-separator interface contributes around 88% of the cell’s total thermal resistance,^20^ while the case-separator interface also gives significant resistance.^21^ However, the thermal resistance at the battery case-coolant interface for immersion cooling systems is yet to be quantified and may be a factor limiting heat removal from the system. Beyond immersion cooling, the iron oxide/alkane interface plays a significant role in other applications such as fuels,^22^ lubricants,^23−25^ and nanofluids.^26^ Nanoparticles suspended in a hydrocarbon fluid can enhance thermal performance,^27^ but the ITR at the nanoparticle-fluid interface is often the bottleneck for heat transfer efficiency.^28,29^ Similarly, in tribological systems where thin lubricant films separate sliding metal surfaces, the ITR can significantly affect heat dissipation.^23−25^ Understanding the heat transfer mechanisms at the iron oxide/alkane interface is therefore crucial for improving thermal management across these diverse applications.

Despite its high relevance to a broad range of applications, the ITR has not been reported for the iron oxide/hydrocarbon interface. This can be attributed, in part, to the absence of a comprehensive force field evaluation for this interface. Many force fields have been tested for the thermal properties of the individual components, hydrocarbons^30−32^ and iron oxide.^33^ Although many iron oxide/hydrocarbon solid–liquid potentials have been proposed in the literature,^23,34−36^ none of these has been verified as suitable for calculations of the ITR through comparison to first-principles methods or experiments. This is an important consideration since the strength of the interfacial interactions strongly affect the wettability and ITR.^37,38^

In this study, we use NEMD simulations to compare the performance of several force fields for their ability to reproduce the key thermophysical properties of hydrocarbons and iron oxide. We then calculate the ITR for the iron oxide/hydrocarbon interface using NEMD simulations with a range of solid–liquid interaction potentials. Finally, we determine the most suitable interaction potential by performing work of adhesion calculations, which are validated against values derived from contact angle experiments. This study represents an important step toward improving the understanding and modeling of nanoscale heat transfer at solid/liquid interfaces, with broad implications for applications such as lubricants, nanofluids and thermal management technologies.

## Methodology

### Liquid Thermal Conductivity

In this study, we use poly-α-olefin (PAO) as a dielectric cooling fluid. PAO is a suitable candidate for battery immersion cooling systems due to its low cost, low toxicity, and adequate working temperature range.^18^ We simulated the branched hydrocarbon hydrogenated 1-decene trimer, which is the main component of PAO4, a synthetic hydrocarbon with approximately 4 cSt viscosity at 100 °C.^24,39^ The molecular structure of PAO4 is shown in Figure S1 of the Supporting Information.

The fluid regions of our systems were generated using the Materials and Process Simulations platform (MAPS) from Scienomics SARL (Paris, France). In order to identify a valid model for the fluid, a series of benchmark simulations were performed using different force fields, with each being assessed on its ability to predict the density, thermal conductivity, and viscosity of the fluid. Four force fields were selected for this investigation, three all-atom (LOPLS-AA,^40,41^ CHARMM36,^42^ AMBER^43^) and one united-atom (TraPPE-UA).^44^ MD simulations were performed in LAMMPS^45^ using a velocity-Verlet integrator.^46^ For the all-atom force fields, a time step of 0.5 fs was used, while for the united-atom force field this was increased to 5 fs. Note that satisfactory energy conservation was not observed for the all-atom force fields with a time step of 1 fs. We did not constrain the C–H bonds with the SHAKE algorithm to account for the full energy transfer along the polymer chain. We used a Lennard-Jones (LJ) cutoff of 12 Å for all of the force fields. The LJ cross interactions were calculated using arithmetic mean mixing rules for CHARMM36 and AMBER, while the geometric mean (Lorentz–Berthelot) mixing rules were used for LOPLS-AA and TraPPE-UA. The long-range Coulombic interactions between the partial charges on the carbon and hydrogen atoms in the all-atom force fields were calculated using the particle–particle particle-mesh (PPPM) algorithm^47^ with a relative error in the forces of 1 × 10^–5^. Intramolecular 1–2 and 1–3 LJ and Coulombic interactions are set to 0.0 for all of the force fields. Intramolecular 1–4 LJ and Coulombic interactions are set to 0.5 for LOPLS-AA and 0.0 for CHARMM36 and TraPPE-UA. For AMBER, the 1–4 LJ interactions are set to 0.5 and the 1–4 Coulombic interactions are set to 0.833.

To calculate the thermal conductivity, a box was created with periodic boundary conditions in all three directions, containing 400 PAO4 molecules at an initial density of 0.8 g cm^–1^. The systems were energy minimized, followed by a 1 ns equilibration in the isobaric–isothermal (NPT) ensemble at 300 K and a pressure of 1 atm. We used the Nosé–Hoover thermostat and barostat^48−50^ with temperature and pressure damping coefficients of 0.1 and 1.0 ps, respectively. Next, the systems underwent a 0.25 ns equilibration in the canonical (NVT) ensemble at 300 K using a Nosé–Hoover thermostat.^48,49^ NEMD simulations were then performed to calculate the thermal conductivity by applying a heat flux along the *z* axis using a boundary driven approach.^51^ Hot and cold thermostat regions were defined in the *xy* plane, with a thickness of 5 Å, at the center and edges of the simulation box. Atoms falling within these regions were thermostated to 325 and 275 K for the hot and cold regions respectively, using the Langevin thermostat.^52^ A temperature damping coefficient of 1.0 ps was used, which ensured good energy conservation during then NEMD simulations.^53^ The NEMD simulations were performed for 5.5 ns, with the initial 0.5 ns discarded to allow the system to reach a nonequilibrium steady state.

To calculate the temperature profile, the system was divided into 200 bins along the *z*-axis and the temperature within each bin (*b*) was calculated using the equation:
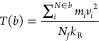
2where *m*_*i*_ and *v*_*i*_ are the mass and velocities of a particle *i* within the bin and *N*_*f*_ is the total number of degrees of freedom in the bin. Since the force fields used in this investigation were fully flexible, *N*_*f*_ = 3*N*_*b*_, where *N*_*b*_ was the number of atoms in each bin.^54^ The temperature profile was calculated every 0.5 ps and averaged across the whole simulation. ∇*T*, was calculated by fitting the temperature profile in the linear regions between the hot and cold thermostats. The heat flux was then calculated according to the equation:
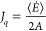
3where ⟨*Ė*⟩ is the time averaged value of the heat rate, , where *E*_*hot*_ and *E*_*cold*_ are the cumulative energies added and subtracted from the atoms in the hot and cold regions during the NEMD simulation respectively, *t* is the time over which the simulation proceeded and *A* is the cross sectional area of the simulation box in the *xy* plane. ⟨*Ė*⟩ was determined by the gradient of the energy exchanged in the hot and cold thermostats against time. The thermal conductivity of the fluid was then calculated using Fourier’s law:^51^
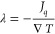
4

Figure S5 in the Supporting Information illustrates a typical temperature profile along the *z*-axis for the PAO4 systems during the NEMD simulation. The system was divided into 200 bins along the *z*-axis and each point on the graph represents the temperature of one bin, averaged over the final 5 ns of the NEMD simulation. ∇*T* was calculated by linear fitting a 10 Å region in the center of the hot and cold thermostats to avoid fitting the nonlinear regions of the temperature profile near the thermostats.^30^ ∇*T* was taken as the average of the two fitted regions. The energies exchanged in the hot and cold thermostats during the simulation are shown by the red and blue lines respectively in Figure S6 in the Supporting Information. The sign of the energy exchanged in the hot thermostat has been flipped to enable comparison of the energy exchanged at both thermostats. By determining the gradient of the two lines and taking the average of the hot and cold thermostats, we obtained the rate of heat exchange, ⟨*Ė*⟩, which can be used in eq 3 to calculate the heat flux.

To check for possible finite size effects in our systems, the simulation box was replicated in the *z* axis, parallel to the heat flux, and the thermal conductivity was recalculated. We found no significant difference in thermal conductivity, indicating that the results were size independent and therefore a 70 × 70 × 70 Å^3^ simulation box was sufficiently large enough to directly obtain the bulk thermal conductivity.

### Liquid Viscosity

To calculate the viscosity, we employed the Green–Kubo method,^55,56^ which relates the shear viscosity of a fluid to the autocorrelation of the off-diagonal elements of the pressure tensor:
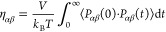
5where η_*αβ*_ is the shear viscosity for components α, β, *V* and *T* are the system volume and temperature, *t* is the time, *P*_*αβ*_ is the off diagonal component of the pressure tensor and ⟨*P*_*αβ*_(0)·*P*_*αβ*_(*t*)⟩ is the pressure autocorrelation function (PACF). There are three off diagonal components of the pressure tensor; *P*_*xy*_, *P*_*xz*_, *P*_*yz*_ and three corresponding viscosity components; η_*xy*_, η_*xz*_, η_*yz*_. The shear viscosity, η, was taken as the average of the 3 η_*αβ*_ components.

The viscosity simulations were performed using a smaller box, containing 100 PAO4 molecules, since previous MD studies have demonstrated a weak system size-dependence using the Green–Kubo method.^57,58^ Periodic boundary conditions were applied in all directions and the systems underwent the same equilibration procedure as described for the thermal conductivity calculations. The equilibrated systems then ran in the NVT ensemble for 50 ns, during which time the integral of the PACF was calculated to obtain the viscosity, using eq 5. The viscosity was obtained by calculating the mean of the viscosity profile once it had converged. The autocorrelation was calculated with a sample rate, *s*, of 10 fs, correlation length, *p*, of 100,000 fs over a correlation time period, *d* = *sp* = 1000 ps. For an explanation of how the correlation time, *d*, was determined, see the Supporting Information and Figure S4.

### Solid Thermal Conductivity

In this study, we have chosen to use iron oxide surfaces to represent the oxidized, outermost layer of the steel casing of a lithium ion battery.^19^ We study hematite (α-Fe_2_O_3_), the most abundant and thermodynamically stable iron oxide under atmospheric conditions. The crystal structure of hematite features layers of oxygen atoms that are hexagonally close-packed and distorted, separated by an iron (Fe^3+^) double layer. Due to its high thermodynamic stability, we investigate the α-Fe_2_O_3_(0001) surface with a half-metal termination (Fe–O_3_-Fe).^59^ In reality, the steel casing will be covered by a heterogeneous mixture of several oxides. However, such spatial heterogeneity cannot be reproduced due to the scale limitations of MD simulations, which means that α-Fe_2_O_3_(0001) is commonly used as a model for steel^23,34,60^ We consider only atomically smooth surfaces. Previous NEMD simulation have shown that for strong solid–liquid interactions, increasing the nanoscale surface roughness has only a minor effect on the ITR.^61^

The surfaces used in this study were generated using the Atomic Simulation Environment (ASE).^62^ To identify the most suitable potential for the solid, a series of benchmark simulations were performed using different force fields, with each being assessed on its ability to predict the density and thermal conductivity of the iron oxide slab. We compared the use of four different potentials, a harmonic model developed by Savio et al.,^34^ a version of ClayFF^35^ with the modifications suggested by Kerisit^63^ to account for the octahedral coordination of iron in hematite, INTERFACE-FF,^36^ and a model developed by Pedone et al.^64^ and later modified by Severin et al.^33^ All of the solid potentials have Coulombic interactions between the iron and oxygen atoms. ClayFF, INTERFACE-FF and the model developed by Savio et al.^34^ employ a LJ potential to describe attractive van der Waals (vdW) interactions and short-range repulsive forces, while the model developed by Severin et al.^33^ uses a Morse potential to characterize these interactions. Additionally, the harmonic model introduces a harmonic bond potential to describe the atomic bonds. These simulations were performed in LAMMPS^45^ using a velocity-Verlet integrator and a time step of 0.5 fs. This was short enough to ensure good energy conservation.^65^ LJ and Morse interactions were cut off at 12 Å and long-range electrostatic interactions were evaluated using the particle–particle particle-mesh (PPPM) solver^47^ with a relative error in the forces of 1 × 10^–5^. LJ cross interactions between were calculated using the geometric mean mixing rules.

To calculate the thermal conductivity of the hematite slabs, we employed boundary-driven NEMD simulations, similar to the ones outlined in the previous section for PAO4. However, when conducting MD simulations involving solid structures, the system size is typically constrained so that the simulation length parallel to the heat flux, *l*_*z*_, is comparable to or smaller than the phonon mean free path (MFP). This can result in the emergence of finite size effects. The MFP represents the average distance a phonon travels before colliding with other energy carriers. In small systems, incomplete phonon scattering events and boundary reflections lead to under predictions in thermal conductivity estimates, compared to “bulk” systems.^66,67^ Therefore, it was necessary to simulate a range of system lengths, allowing us to apply the linear extrapolation method to determine the “bulk” thermal conductivity.^33,68^ This approach can lead to underestimation of the bulk thermal conductivity when *l*_*z*_ is relatively short (compared to the average phonon MFP).^65,69^

Initially, a 50.3 × 43.6 × 54.9 Å^3^ hematite slab was generated, which was then replicated in the *z* direction, parallel to the [100] crystallographic direction, to create additional systems of length 109.8, 164.8, 219.7, and 274.6 Å. The systems were energy minimized, followed by a 1 ns equilibration in the NPT ensemble at 300 K with an anisotropic pressure coupling of 1 atm in the *z*-axis, using a Nosé–Hoover thermostat and barostat^48−50^ with temperature and pressure damping coefficients of 100 fs and 1,000 fs, respectively. Finally, the system underwent a 0.25 ns equilibration in the NVT ensemble at 300 K using a Nosé–Hoover thermostat.^48,49^ Once the system had equilibrated, NEMD simulations were performed to calculate the thermal conductivity by applying a heat flux along the *z*-axis. Hot and cold thermostat regions were defined in the *xy* plane, with a thickness of 5 Å, at the center and edges of the simulation box. Atoms falling within these regions were thermostated to 325 and 275 K for the hot and cold regions respectively, using a Langevin thermostat.^52^ Previous comparisons suggested that the Langevin thermostat is preferrable to the Nosé–Hoover thermostat for this purpose.^65^ NEMD simulations were performed for 3 ns, with the initial 0.5 ns being discarded to allow the system to reach a nonequilibrium steady state. The temperature gradient, ∇*T*, and heat flux, *J*_*q*_, were calculated using method outlined previously, allowing the size dependent thermal conductivity to be calculated.

Figure S5 in the Supporting Information illustrates a typical temperature profile along the *z*-axis for the Fe_2_O_3_ systems during the NEMD simulations. The system was divided into 20*n* bins, where *n* was the number of times the initial 50 Å simulation was replicated in the *z* axis. The points on the graph represent the temperature of each bin, averaged over the final 5 ns of the simulation. As the box length, *l*_*z*_, increased, so did the size of the nonlinear regions by the thermostats and as such, an exclusion zone of 5*n* was left on either side of the thermostats when calculating the temperature gradient to avoid fitting in the nonlinear region.^65^ The heat flux was obtained by calculating ⟨*Ė*⟩ at the hot and cold thermostats and the thermal conductivity was calculated using Fourier’s law. In all cases investigated here, there was a < 1% difference in the total energy exchanged at the hot and cold thermostats during NEMD simulations, indicating good energy conservation.^65^

To calculate the bulk thermal conductivity, κ_∞_, the inverse of the thermal conductivity, , was plotted against the inverse of the system length, ,^33,68^ as displayed for each force field in Figure S6 in the Supporting Information. For ClayFF, INTERFACE-FF and the Morse potential, all system sizes were found to fall within the linear regime; however, for the harmonic model only the systems between 100 and 250 Å fell in the linear regime. The linear regime was fitted and extrapolated to obtain the *y* intercept, which corresponds to . The thermal conductivity was also calculated in the [001] and [010] directions to investigate the anisotropy of this property in hematite.^33^

### Interfacial Thermal Resistance

Using the most suitable liquid force fields and solid potentials identified in the previous sections, we performed simulations of the α-Fe_2_O_3_–PAO4 interface to calculate the ITR. The initial starting configuration contained a 50.29 × 43.55 × 54.92 Å^3^ hematite surface, generated using ASE,^62^ placed between two liquid regions, containing a total of 440 PAO4 molecules. To check for possible finite size effects, the length of the PAO4 and hematite regions were varied in the direction parallel to the heat flux and *R*_*k*_ was computed. The results showed no size dependency and it was therefore assumed that the system described above was sufficiently large enough to calculate the “bulk” ITR.

The solid–liquid interactions were defined by a potential, *U*_*sl*_, which encompasses both LJ and Coulombic interactions:
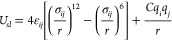
6The first term defines the LJ forces, where ε_*ij*_ represents the strength of the interaction, σ_*ij*_ represents the distance at which the potential between particles *i* and *j* is zero and *r* represents the separation between the particles. The second term defines the Coulombic interaction, where *C* represents an energy conversion constant and *q*_*i*_ and *q*_*j*_ represent the charges on atoms *i* and *j*.

The LJ parameters between two dissimilar atoms, ε_*ij*_ and σ_*ij*_, were calculated using the geometric mean mixing rules:
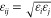
7
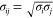
8

A wide range of different LJ parameters for interactions between hematite and various fluids can be found in the literature. Since none of these have been verified for the nanoscale thermal transport, we systematically vary the LJ potential parameters for the hematite atoms, ε_*Fe*/*O*_ and σ_*Fe*/*O*_, drawing from different sources, including; ClayFF,^35^ INTERFACE-FF,^36^ Berro et al.^23^ and Savio et al.^34^ These parameters were combined, using the geometric mean mixing rules, with the LJ parameters for the fluid, ε_*C*/*H*_ and σ_*C*/*H*_, which were taken from the LOPLS-AA force field.^40,41^ In total, we compared eight different LJ potentials, with varying solid–liquid interaction strength. The Fe and O LJ parameters used for each of the solid–liquid potentials are shown in Table 1. Full LJ parameters for solid–liquid interactions are shown in Table S1 in the Supporting Information. Previous comparisons have suggested that, compared to ClayFF,^35^ the stronger LJ interactions proposed by Savio et al.^34^ more accurately reproduced force–distance curves obtained from DFT for hydrocarbons adsorbed on hematite surfaces.^60^

**Table 1:** LJ Parameters for the Fe and O Atoms Used for the Different Surface–Fluid Interfacial Force Fields

	ε/kcal mol^–1^		σ/Å	
Potential	Fe	O	Fe	O
ClayFF^35,63^	9.0 × 10^–6^	0.1554	4.0720	3.1660
Berro_0.5 ε_*Fe*__^23^	0.0850	0.1699	2.2000	2.9600
Berro_1.0 ε_*Fe*__^23^	0.1699	0.1699	2.2000	2.9600
INTERFACE-FF^36^	0.3000	0.2000	1.8500	3.1270
Berro_2.5 ε_*Fe*__^23^	0.4248	0.1699	2.2000	2.9600
Berro_7.5 ε_*Fe*__^23^	1.2743	0.1699	2.2000	2.9600
Berro_15.0 ε_*Fe*__^23^	2.5485	0.1699	2.2000	2.9600
Savio^34^	9.5180	0.2100	2.3210	2.9600

To ensure the stability of the surfaces using the solid Morse potential, the partial charges remained constant for all of the interfacial force fields at *q*_*Fe*_ = +1.800 and *q*_*O*_ = −1.200.^33,64^ This also meant that any differences in the ITR were due to changes to the LJ parameters. Note that the original INTERFACE-FF (*q*_*Fe*_ = +1.740, *q*_*O*_ = −1.160),^36^ ClayFF^35,63^ (*q*_*Fe*_ = +1.575, *q*_*O*_ = −1.050), and Berro et al.^23^ (*q*_*Fe*_ = +0.771, *q*_*O*_ = −0.514) force fields used smaller partial charges. Tests were also performed for ClayFF^35,63^ using the original LJ parameters for the solid–solid interactions and partial charges. For the relatively nonpolar PAO molecules considered here, we found that the ITR was identical to that obtained with the modified partial charges within the uncertainty of the NEMD simulations. These comparisons are shown in Figure S7 in the Supporting Information.

The NEMD simulations to calculate the ITR were performed in LAMMPS^45^ using a velocity-Verlet integrator and a time step of 0.5 fs. This time step was sufficiently short to ensure energy conservation during the NEMD simulations.^53^ Five independent repeats were performed for each of the eight solid–liquid potentials, with each trajectory being given its own randomly generated initial velocity seed. Each system was energy minimized, followed by a 1 ns equilibration in the NPT ensemble at 300 K with an anisotropic pressure coupling of 1 atm in the *z*-axis, using a Nosé–Hoover thermostat and barostat^48−50^ with temperature and pressure damping coefficients of 0.1 and 1.0 ps respectively. Temperature, energy and density data are plotted during the NPT equilibration stage in Figure S8 in the Supporting Information. Finally, the systems underwent a 0.25 ns equilibration in the NVT ensemble at 300 K using a Nosé–Hoover thermostat.^48,49^ To calculate the ITR, a heat flux was applied along the *z*-axis, as described in the previous sections. The hot and cold thermostats regions had a thickness of 10 Å and atoms falling within these regions were thermostated to 325 and 275 K respectively, using a Langevin thermostat^52^ with a temperature damping coefficient of 1.0 ps. Previous studies have highlighted the importance of appropriate thermostatting when calculating the ITR from NEMD simulations.^53,70^ In particular, for stochastic thermostats such as Langevin, if the coupling of the thermostat and the system is too strong (short temperature damping coefficient), then spurious temperature jumps can be created between the solid and fluid regions, leading to overestimated ITR.^53,70^ Therefore, we ensure that our choice of temperature damping coefficient was sufficiently large such that it did not influence our ITR results.

The NEMD simulations were performed for 5.5 ns, with the initial 0.5 ns discarded to allow the system to reach a nonequilibrium steady state. During the NEMD simulations, the center of mass (COM) of the slab was fixed in the *z*-direction to prevent drift. Temperature and density profiles were calculated by dividing the system into 100 and 5,000 bins, respectively, along the *z*-axis. The temperature within each bin was calculated using eq 2 and the density was calculated using equation, , where *n*_*i*_ is the number of atoms of type *i* in the bin with mass, *m*_*i*_, and *V* was the volume of the bin.

### Vibrational Density of States

The vibrational density of states (VDOS) was calculated for the liquid and solid regions surrounding the interface by performing a Fourier transformation of the velocity autocorrelation function (VACF):
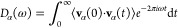
9where ⟨**v**_α_(0)·**v**_α_(*t*)⟩ is the VACF of species α, and ω is the frequency. The VDOS for the fluid, *D*_*PAO*4_(ω), was calculated for all fluid atoms falling within the first adsorption layer and the VDOS for the surface, *D*_Fe_2_O_3__(ω), was calculated for all surface atoms within the two outermost atomic layers. After sampling various correlation times for the VACF, we determined that a correlation time of 4 ps provided the optimal balance, yielding comprehensive vibrational information while minimizing noise in the resulting VDOS. During this time, < 5% of initial interfacial PAO4 molecules had left the region. The same systems as used for the ITR calculations were also used for the VDOS calculations.

### Work of Adhesion

Previous studies have shown that the solid–liquid work of adhesion is proportional to the ITC.^71^ Since the ITR/ITC has not been experimentally quantified for our interface of interest, we use the work of adhesion as a proxy measurement to tune the strength of the solid–liquid LJ interactions. This approach was also used by Leroy et al.^72^ for the graphene–water interface.

The solid–liquid work of adhesion, *W*_*sl*_, defines the reversible work required per unit area to separate the interface between a solid and a liquid phase to an infinite distance. It indicates the relative strengths of adhesive and cohesive forces at the interface. Positive *W*_*sl*_ denotes strong adhesion, leading to wetting, while negative *W*_*sl*_ signifies poor wetting with dominant cohesive forces. Zero *W*_*sl*_ indicates equilibrium between adhesive and cohesive forces, resulting in negligible interaction. The *W*_*sl*_ is given by the Young-Dupré equation:
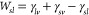
10where γ_*sv*_, γ_*sl*_ and γ_*lv*_ represent the solid–vapor surface tension, solid–liquid surface tension and liquid–vapor interfacial tension acting on the equilibrium point. The combination of Young’s equation, Equation S1 in the Supporting Information, and eq 10 yields the equation for *W*_*SL*_ in terms of the liquid–vapor interfacial tension, γ_*lv*_, and the contact angle, θ:
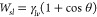
11

A direct approach to calculating *W*_*sl*_ involves using eq 11 and obtaining contact angles through simulations of nanoscale droplets. However, obtaining θ can give rise to a number of complications, such as long equilibration times,^73^ finite size effects^74^ and difficulty calculating contact angles in extreme or fully wetting scenarios, which introduces uncertainty into the results.

Recently, alternative methodologies have been developed to enable the calculation of *W*_*sl*_, without requiring the simulation of nanoscale droplets. Instead these approaches rely on thermodynamic integration. The phantom wall method involves the use of a virtual piston to separate the solid and liquid phases,^75^ while the dry-surface method^76^ involves perturbing the interactions between the solid and liquid phases by introducing a coupling parameter, λ, to transition from a state where interactions are fully coupled, to a fully decoupled state.^76^ In this work we will employ the dry-surface method with damped Coulombic interactions, as proposed by Surblys et al.^77^

To calculate *W*_*sl*_, simulations were performed using the same system as used in previous *R*_*k*_ calculations. The temperature was maintained at 300 K with a global Nosé–Hoover thermostat with a damping coefficient of 0.1 ps.^48,49^ The pressure was kept at 1 atm by applying an anisotropic Nosé–Hoover barostat^48−50^ with a damping coefficient of 1.0 ps in the *z*-direction. It was suggested by Surblys et al.^77^ that systems with long-range Coulombic interactions require the solution of additional Poisson equations when performing dry-surface calculations. As a solution, the long-range Coulombic potential for solid–liquid interactions was replaced with a new potential which made use of damped Coulombic interactions:
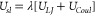
12where λ represents the coupling parameter. *U*_*LJ*_ was given by a 12–6 LJ potential and *U*_*Coul*_ was given by a damped shifted force model, described by Fennel:^78^
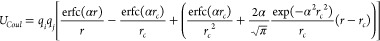
13where *q* was the charge of particles *i* or *j*, *erfc*() is the complementary error-function, α is the damping parameter and *r*_*c*_ is the cutoff distance of Coulombic interactions. The cutoff distance was set to a value of *r*_*c*_ = 12 Å and the damping parameter was set to a value of α = 0.2 Å^–1^. LJ and Coulombic interactions were gradually switched off, by scaling λ from λ = 1 for fully coupled solid–liquid interactions, to λ ≈ 0 for fully uncoupled solid–liquid interactions. The coupling parameter was not sampled at λ = 0 to avoid numerical instability that may arise when solid–liquid interactions are entirely decoupled. Values for λ = 0 were extrapolated from the two nearest data points. The work of adhesion could be obtained using the equation:
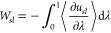
14where *u*_*sl*_ is the solid–liquid potential energy per unit area, given by the equation:
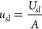
15where *U*_*sl*_ is the solid–liquid potential energy and *A* is the cross-sectional area, *l*_*x*_ × *l*_*y*_. The Coulombic and LJ contributions to *W*_*sl*_ were each obtained using the following equations:
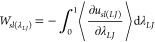
16
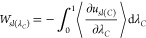
17
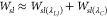
18

The coupling parameter, λ, was sampled at 24 equally spaced data points in the range λ ∈(0,1]. At each point, the system was equilibrated for 0.25 ns followed by a 0.75 ns run to obtain *u̅*_*sl*_. The Hamiltonian derivatives, , were computed using two distinct methods: numerical differentiation and polynomial fitting. The first method employed numerical differentiation of *u*_*sl*_ through the central differences scheme. The second method involved fitting *u*_*sl*_ with a second-order polynomial and subsequently obtaining its derivative. The resulting numerical and polynomial Hamiltonian derivatives were then integrated using the trapezoid rule and analytic methods, respectively, allowing *W*_*sl*_ to be calculated using eqs 16-18. *W*_*sl*_ is presented as the average result of the two differentiation methods. The same systems as used for the ITR and VDOS calculations were also used for the *W*_*sl*_ calculations.

## Results and Discussion

### Liquid Thermal Conductivity

Thermal conductivities calculated using each force field are presented in Table 2. In all cases, the thermal conductivity calculated using the all-atom force fields was overestimated. Using AMBER resulted in a 60% overestimation, LOPLS-AA 53%, and CHARMM-36 46% compared to the experimental value of 0.15 W K^–1^ m^–1^ at room temperature and pressure.^79^ Previous studies have reported that, in general, all-atom force fields overpredict the thermal conductivity of alkane liquids.^30−32^ However, all-atom force fields performed better than united-atom force fields in predicting the thermal conductivity of solid alkanes^80^ and liquid polymers,^81^ indicating the importance of including explicitly the CH_2_ and CH_3_ groups in these cases. It has been speculated that the overprediction of all-atom force fields may be a systematic error related to their inability to capture quantum effects.^82^ In particular, high-frequency degrees of freedom, such as the vibrational modes of hydrogen atoms, can contribute to thermal conductivity in MD simulations when in reality, these modes are typically in their quantum mechanical ground state and do not significantly participate in heat conduction.^80^ This can result in an increase in the apparent number of heat carriers, leading to an overestimation in thermal conductivity. We note, however, that recent computations using rigid and flexible water molecules reveal similar thermal conductivities for these models,^83,84^ indicating that high-frequency modes might not contribute to a significant enhancement of the thermal conductivity. It is also noteworthy that the experimental sample of PAO4 used to calculate the thermal conductivity^79^ will have contained a mixture of isomers of 1-decene dimers, trimers, and tetramers,^39^ rather than the pure trimer used in our MD simulations. These impurities may affect the thermal conductivity, which complicates the validation of the chosen force fields.^31^

**Table 2:** Comparison of Density (ρ), Viscosity (ν), and Thermal Conductivity (κ), Calculated for PAO4 Using Different Force Fields, with Experimental Data

	ρ/g cm^–3^		ν/cSt		κ/W K^–1^ m^–1^
Force Field	313 K	373 K	313 K	373 K	300 K
Experimental	0.80^39^	0.76^39^	16.25^39^	3.74^39^	0.15^79^
LOPLS-AA	0.79 ± 0.00	0.74 ± 0.01	10.4 ± 1.6	3.3 ± 0.9	0.23 ± 0.02
CHARMM-36	0.79 ± 0.00	0.74 ± 0.01	8.4 ± 0.4	2.3 ± 0.3	0.22 ± 0.01
AMBER	0.79 ± 0.00	0.75 ± 0.00	49.4 ± 15.2	8.3 ± 3.6	0.24 ± 0.03
TraPPE-UA	0.78 ± 0.00	0.74 ± 0.00	2.6 ± 0.6	1.2 ± 0.1	0.11 ± 0.01

On the other hand, the thermal conductivity calculated using the united-atom force field, TraPPE-UA, was underpredicted by 27%. United-atom models enhance computational efficiency by removing hydrogen atoms and applying constraints to bonds and angles, thereby reducing the number of degrees of freedom. It is believed that this reduction in degrees of freedom, particularly the high-frequency hydrogen modes, is responsible for the lower thermal conductivity predicted by united-atom models compared to all-atom models,^32,80,85^ but these conclusions need to be considered in the wider context. As noted above rigid and flexible models of water predict rather similar thermal conductivities.^83,84^ These studies indicate that high-frequency modes do not provide a general explanation of the observed behavior. It may be possible to address these deviations from experimental results through careful reparameterisation, however, that is beyond the scope of this current investigation.

### Liquid Viscosity

For each force field, the viscosity was calculated at 313 and 373 K to enable comparison with experimental data. The results are shown in Table 2. The viscosity calculated with the united-atom force field was under predicted by 84% at 313 K and 68% at 373 K with respect to experimentally obtained values of 16.3 and 3.7 cSt at 313 and 373 K, respectively.^39^ This significant under-prediction is consistent with the known limitations of united-atom models, which often fail to accurately predict viscosity.^86^ This issue is thought to arise from the omission of hydrogen atoms and the focus of parametrization on equilibrium properties rather than dynamic liquid properties. For instance, the TraPPE-UA model is parametrized specifically to match saturated liquid density.

Of the all-atom force fields, AMBER performed the worst, resulting in overestimations of 204% at 313 K and 122% at 373 K. This finding was consistent with previous simulations, which suggested that the AMBER model overpredicts the gel to liquid phase transition temperature in long chain hydrocarbons, resulting in the significant overprediction of viscosity at 313 K due to PAO4 being in a semicrystalline state.^86,87^ Similarly, CHARMM-36 under predicted the viscosity by 48% at 313 K and 37% at 373 K. The CHARMM-36 force field, optimized primarily for simulations of phospholipids and biomolecules, is less suited for long-chain alkanes, which likely contributes to its poorer viscosity predictions compared to LOPLS-AA. The LOPLS-AA force field was optimized to reproduce the liquid properties of long chain hydrocarbons. Consequently, it provided the best results for viscosity among the tested force fields, yielding a 36% underprediction at 313 K and a 12% underprediction at 373 K. These results are consistent with previous studies comparing the use of classical force fields to predict viscosity of linear^86^ and branched^88^ alkanes.

Of the force fields tested in this work, we determined LOPLS-AA to be the most suited for further simulations. While all force fields predicted the fluid density well, we saw large differences between simulation and experimental results with for the thermal conductivity and viscosity calculations. While TraPPE-UA made the best predictions for the fluid thermal conductivity, we observed significant deviations from the experimental viscosity at both high and low temperatures. While the three all-atom force fields all showed similar over predictions in thermal conductivity, LOPLS-AA provided significantly better predictions for the viscosity at both high and low temperatures and therefore provides a better model for the key dynamical properties that determine the characteristic dynamic behavior of the liquid. As a result, we chose to use the LOPLS-AA force field to model the fluid for the remainder of simulations in this study.

### Solid Thermal Conductivity

Table 3 presents the calculated densities, ρ, and κ_∞_ in the different crystallographic directions, calculated for hematite at 300 K for each of the different force fields. Dreyer et al.^89^ reported an experimental value of 14.7 W K^–1^ m^–1^ for a single crystal of hematite in the [100] and [010] direction, and 12.1 W K^–1^ m^–1^ in the [001] direction. We found that using the harmonic model yielded a thermal conductivity that was overestimated by >500%. We believe this overestimation to be a result of the harmonic bond potential, which assumes that atoms oscillate around their equilibrium positions in a perfectly harmonic manner. In reality, solids exhibit anharmonic behavior. Anharmonicity impedes thermal conductivity in crystalline solids and therefore the failure of this model to capture that behavior results in overestimations of thermal conductivity. The three remaining models were used to calculate the thermal conductivity, with ClayFF underestimating and INTERFACE-FF underestimating the thermal conductivity by 71% and 47% respectively, and the Morse potential giving a thermal conductivity within 1% of the experimental value. All three models gave thermal conductivities that were significantly lower than the results of the covalent model due to the absence of the harmonic bond potential. ClayFF and INTERFACE-FF underestimated the thermal conductivity because of the use of a LJ potential to describe the vdW interactions, which provides a less accurate representation of the nonbonded interactions than the Morse potential used in the other model.^33^ As the thermal conductivity of Fe_2_O_3_ is anisotropic,^89^ it was further investigated in the [010] and [001] directions using the Morse potential. We obtained a value of 12.14 and 12.22 W K^–1^ m^–1^ in the [010] and [001] directions respectively, which were within 1% of the experimentally determined values. From these results, we conclude that the Morse potential was the most suitable model for our Fe_2_O_3_ surface and will therefore be used in all further simulations.

**Table 3:** Comparison of Density (ρ) and Thermal Conductivity (κ), Calculated for Hematite at 300 K and 1 atm Using Different Force Fields, with Experimental Data

		κ/W K^–1^ m^–1^		
Force Field	ρ/g cm^–3^	[100]	[010]	[001]
Experimental	5.3^90^	14.7^89^	12.1^89^	12.1^89^
Morse^33^	5.2 ± 0.1	14.5 ± 0.2	12.2 ± 0.2	12.2 ± 0.1
INTERFACE-FF^36^	5.3 ± 0.1	6.5 ± 0.2	-	-
ClayFF^35,63^	5.3 ± 0.1	4.3 ± 0.1	-	-
Harmonic^34^	5.3 ± 0.1	93.8 ± 9.2	-	-

### Interfacial Thermal Resistance

**Figure 1:**
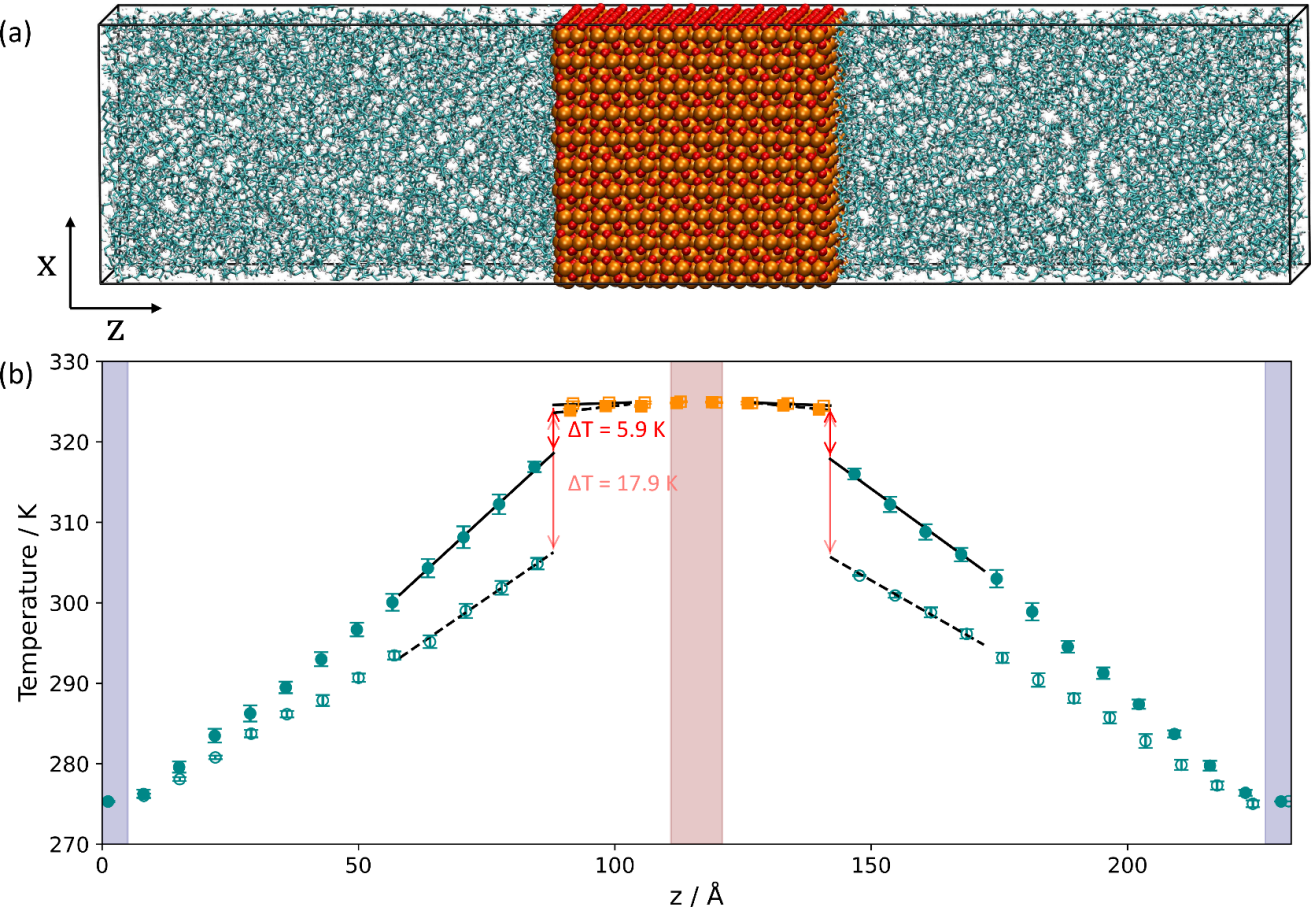
(a) System used to compute the interfacial thermal resistance of the α-Fe_2_O_3_–PAO4 interface. The snapshot was visualized in VMD.^91^ (b) Time-averaged temperature profiles of the PAO4 fluid (cyan markers) and Fe_2_O_3_ slab (orange markers) obtained from NEMD simulations. Examples shown for the strongest (unfilled markers) and weakest (filled markers) solid–liquid potentials. Linear fits were obtained in the regions near the interface and extrapolated to the interface, allowing the temperature drop, *ΔT*, to be calculated (illustrated by red arrows). The red and blue shaded regions represent the positions of the hot and cold thermostats, respectively. The error bars represent the standard deviation over five independent simulations.

Using the LOPLS-AA force field^40,41^ to capture fluid behavior and the Morse potential^33^ to represent surface behavior, we computed the ITR for each of the eight solid–liquid LJ potentials. Figure 1 shows the starting configuration used for these calculations and the time-averaged temperature profiles obtained for the weakest (ClayFF^35,63^) and strongest (Savio et al.^34^) solid–liquid LJ potentials. The temperature points near the thermostat and interface regions have been ignored in the linear fits for the solid and liquid temperature profiles.^4^ The linear fits were then extrapolated to the interface to calculate the temperature jump, *ΔT*. For ClayFF,^35,63^*ΔT* = 17.9 K, while for Savio et al.^34^*ΔT* = 5.9 K.

**Figure 2:**
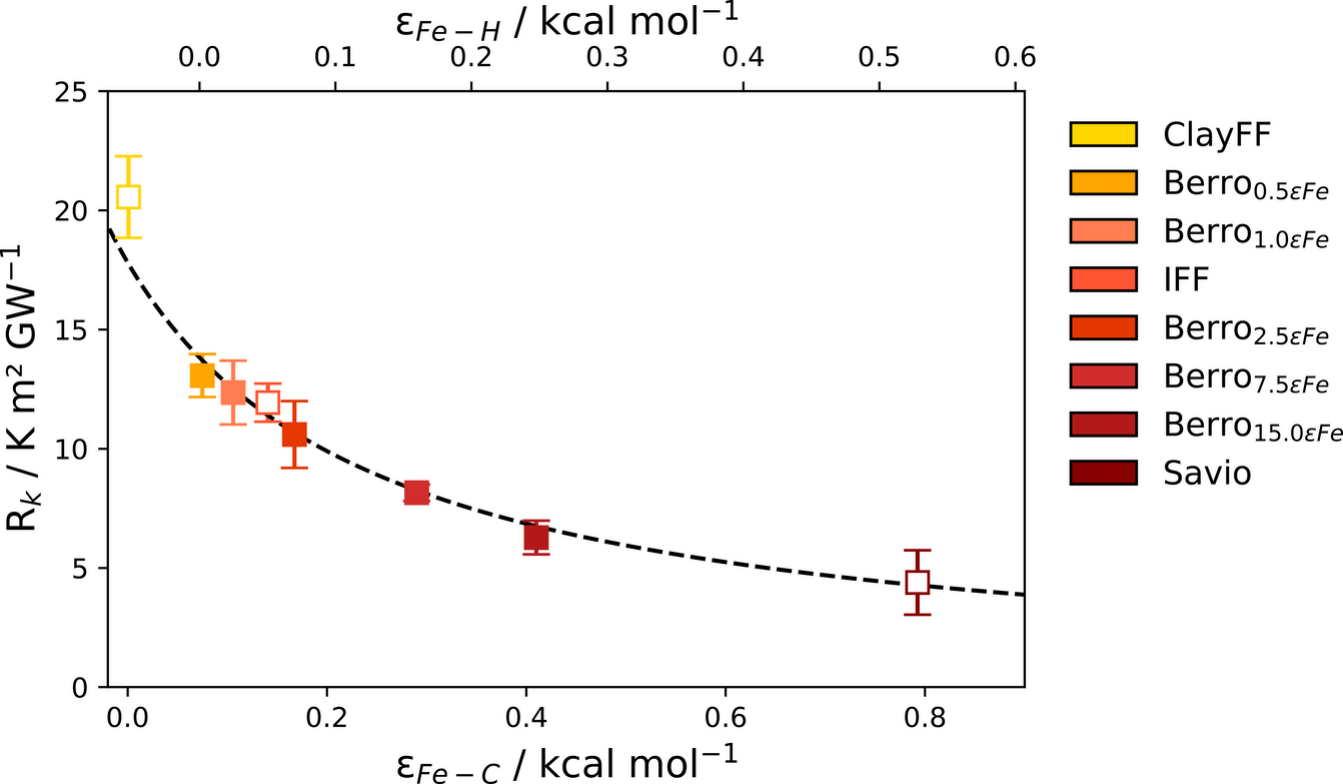
Interfacial thermal resistance (ITR) plotted as a function of the LJ well-depth parameter for iron–carbon interactions, ε_*Fe*–C_ (lower axis), and iron–hydrogen interactions, ε_*Fe*–H_ (upper axis). The dashed black line is a linear fit of *R*_*k*_^–1^ versus ε_*Fe*–C_. Filled markers represent potentials where only ε_*Fe*–PAO_ was changed. Unfilled markers ε_*Fe*–PAO_ represent potentials where ε_*O*–PAO_ and σ parameters were also changed.

Figure 2 shows the change in ITR with the ε LJ parameter for the Fe–C, ε_*Fe*–C_, and Fe–H, ε_*Fe*–H_, interactions. As the strength of these interactions was increased, the ITR decreased from *R*_*k*_ = 20.6 ± 1.7 K m^2^ GW^–1^ for ClayFF^35,63^ to *R*_*k*_ = 4.4 ± 1.4 K m^2^ GW^–1^ for Savio et al.^34^ The value calculated for the strongest potential is similar to that reported from previous NEMD simulations of the lower limit of the ITR (*R*_*k*_ ∼ 6 K m^2^ GW^–1^) for ionic liquid-graphene interfaces.^7^ The range of ITR values is also similar to that observed experimentally (*R*_*k*_ = 6–20 K m^2^ GW^–1^) for the interface between water and chemically functionalized aluminum and gold surfaces.^92^

We also calculate the so-called Kapitza length,^38^ which suggest that the ITR will be important to overall heat transfer over length scales corresponding to *l*_*K*_ = *κ G*_*K*_ ∼ 1–10^2^ nm for the hematite/PAO interface, depending on the solid/liquid interaction strength. For comparison, previous MD simulations suggested that the Kapitza length for the silicon/water interface was 6 nm.^93^ While these results suggest that ITR is unlikely to be the limiting factor for heat dissipation in immersion cooling at current macroscopic length scales, these length scales are relevant in nanoengineered surfaces, which are attracting attention in various thermal management applications, such as immersion cooling in data centers.^94^ If these surfaces are incorporated into future EV battery immersion cooling systems, minimizing ITR at these interfaces could become necessary for optimizing nanoscale heat management. Additionally, ITR will be a critical factor in other nanoscale applications such as nanofluid coolants,^27^ where ITR at the nanoparticle-fluid interface is often a bottleneck for heat transfer efficiency.^28,29^

Most previous NEMD simulation studies have studied the relationship between the strength of the solid–liquid LJ interactions and the ITC, rather than the ITR. In our NEMD simulations, the ITC was found to increase linearly with ε_*Fe*–C_ and ε_*Fe*–H_, as shown in Figure S9 in the Supporting Information. This linear relationship between *G*_*k*_ and ε has been reported in previous NEMD studies for simple LJ systems^95,96^ and the Si/Water interface.^15^ We do not observe an exponential scaling of *G*_*k*_ with ε_*Fe*–C_ and ε_*Fe*–H_, as has been observed in some NEMD simulations at low interaction strengths.^95,96^

We conducted further analysis to explore the temperature dependency of the ITR. Simulations were carried out at a range of temperatures, *T* = 250–500 K, where *T* represents the mean temperature of the hot and cold thermostats. This temperature range was selected to sample the full liquid range of PAO4. Our findings revealed that there was no significant variation in ITR across the range of temperatures tested. Detailed results of these investigations are presented in Figure S10 in the Supporting Information.

### Vibrational Density of States

**Figure 3:**
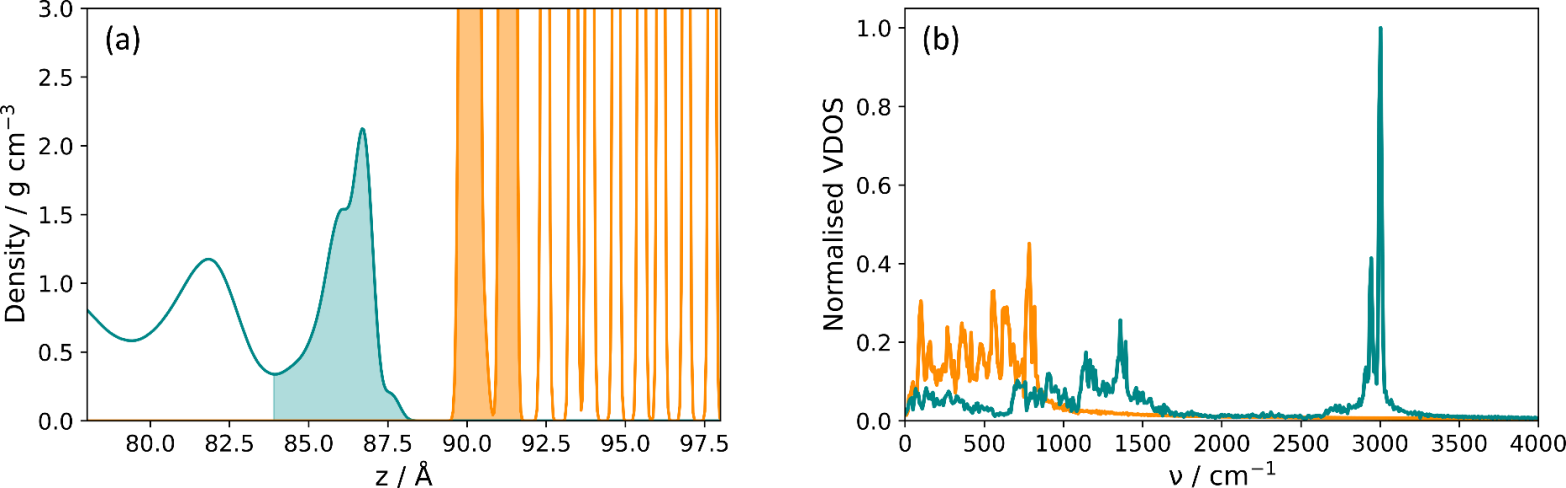
(a) Density profile of PAO4 (cyan) and hematite (orange) near the interface. The shaded regions under each curve represent the regions from which the vibrational density of states (VDOS), *D*_*PAO*4_(ω) and *D*_*Fe*_2_*O*_3__(ω) were calculated. (b) Corresponding VDOS for PAO4 (cyan) and Fe_2_O_3_ calculated for the regions shaded in (a).

To investigate molecular-level origins of the observed dependence of the ITR on the solid–liquid interaction strength, we calculated the VDOS for the α-Fe_2_O_3_ and PAO4 regions adjacent to the interface for potentials each potential. The interfacial regions used to calculate the VDOS are illustrated in Figure 3 (a) and the resulting VDOS is shown in Figure 3 (b). The VDOS of PAO4 showed peaks at ν = 700, 900, 1160 cm^–1^, which corresponded to C–C stretching, ν = 1340 cm^–1^, which corresponded to C–H bending, and ν = 3000 cm^–1^, corresponding to C–H stretching.^97^ The VDOS for α-Fe_2_O_3_ showed a series of peaks between 100 and 800 cm^–1^, which is consistent with previous experimental studies for hematite.^98^

**Figure 4:**
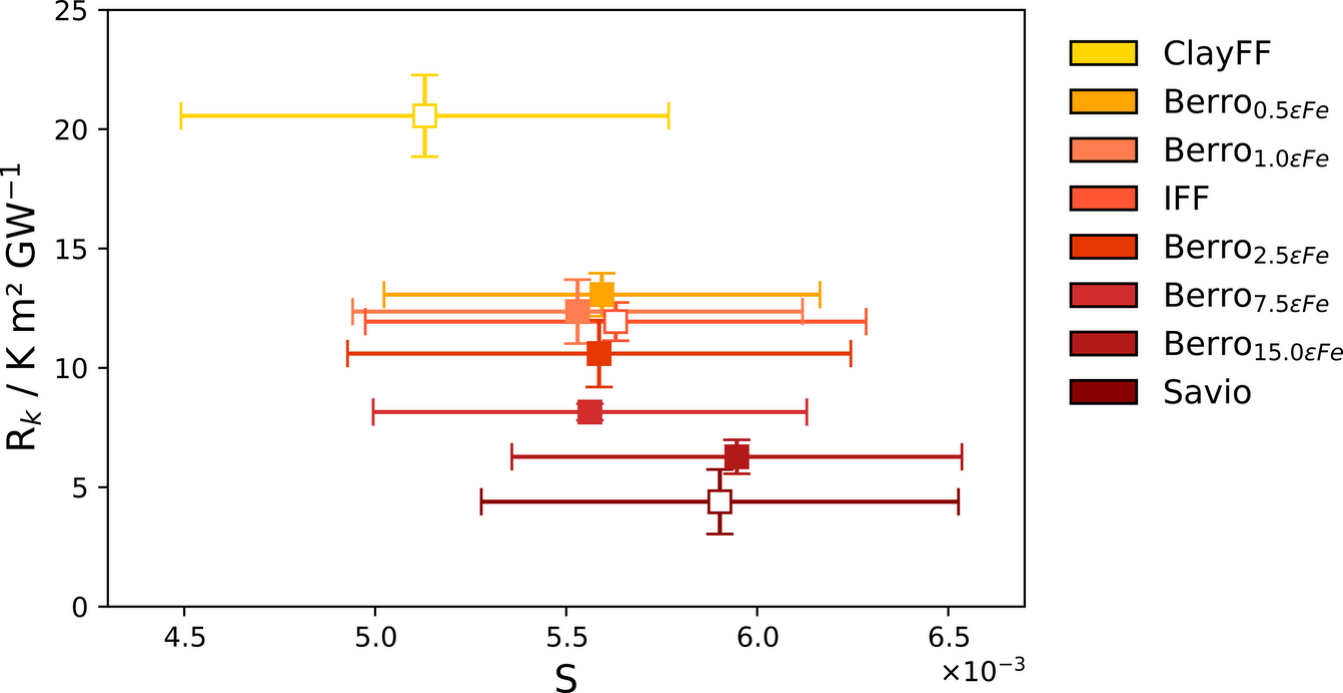
ITR plotted as a function of the VDOS overlap integral, *S*. Filled markers represent potentials where only ε_Fe–PAO_ was changed. Unfilled markers ε_Fe–PAO_ represent potentials where ε_O–PAO_ and σ parameters were also changed.

Figure S11 in the Supporting Information shows the VDOS calculated for the of the interfacial α-Fe_2_O_3_ and PAO4 regions. Upon initial inspection there appeared to be no change as the solid–liquid interaction strength was increased. However, closer inspection revealed a reduction of the low frequency transverse modes as interaction strength was increased, indicating an enhanced coupling of the transverse modes.^99^ This allows for more efficient energy exchange between the solid and the liquid phases and therefore, a reduction in the ITR. In an attempt to quantify this increase in coupling, we calculated the overlap of the VDOS for α-Fe_2_O_3_ and PAO4:^100,101^
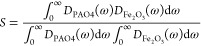
19where *D*_PAO4_ and *D*_Fe_2_O_3__ are the VDOS for the PAO4 and Fe_2_O_3_ regions, respectively. *S* has been used in a number of studies to rationalize changes to the ITR of solid–liquid and solid–solid interfaces.^100,101^ We calculated *S* specifically for low-frequency modes, focusing on the range from 0 to 200 cm^–1^, as changes were only observed within this frequency range. Figure 4 shows the ITR plotted as a function of S. We observe a trend that as *S* increases the ITR is reduced. However, due to significant overlap in the error bars, this trend is not statistically significant. These findings were consistent with results reported by Alexeev et al.,^102^ who found no correlation between *R*_*k*_ and the VDOS overlap, *S*, for the water-graphene interface. Given the lack of significant change in S, it is likely that other factors beyond the low-frequency modes are influencing the observed trend in ITR.

### Interfacial Liquid Structure

We observed a significant change in the density profiles of PAO4 for the different solid–liquid potentials. In all cases, the PAO4 density profile exhibits oscillatory behavior in the region near the interface. This behavior arises from the organization of PAO4 molecules into layers near the surface, due to the strong intermolecular interactions between PAO4 molecules and the Fe_2_O_3_ surface. The first adsorption layer, situated nearest to the Fe_2_O_3_ surface, displays the highest density and is followed by a series of peaks of diminishing density as the distance from the surface is increased. At a distance of ∼25 Å from the surface, the density profiles level and approach the bulk liquid density (0.8 g/cm^3^).^39^ The layering of PAO4 molecules near the interface did not significantly affect the fluids thermal conductivity, as shown by the consistent temperature gradient observed in both the bulk and interfacial liquid regions in Figure 1. This finding aligns with previous NEMD simulations that examined the effect of liquid ordering on heat flow in an atomic LJ solid–liquid system.^95^

**Figure 5:**
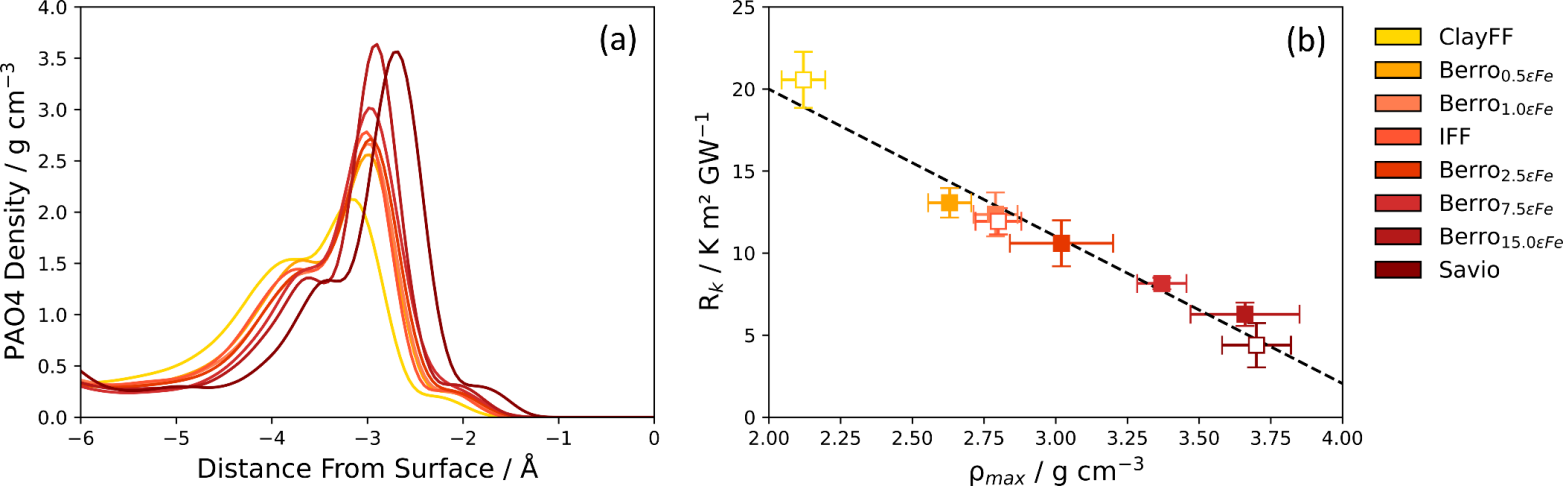
(a) PAO4 density profile for the region adjacent to the left side of the PAO4/hematite interface, shown for each solid–liquid potential. The *z* coordinate represents the distance from the hematite surface. (b) Peak density of PAO4 in the first adsorption layer, *p*_*max*_. The dashed black line is a linear fit of *R*_*k*_ versus *p*_*max*_. Filled markers represent potentials where only ε_Fe–PAO_ was changed. Unfilled markers ε_Fe–PAO_ represent potentials where ε_O–PAO_ and σ parameters were also changed.

The peak density of the first adsorption layer, ρ_*max*_, was found to increase with the strength of the solid–liquid LJ potential, while the average density in the bulk region of the liquid layer remained unchanged, Figure 5 (a). We observed a strong dependence of *R*_*k*_ on ρ_*max*_, with a higher density resulting in a smaller *R*_*k*_, as shown in Figure 5 (b). This relationship was first observed by observed by Challis et al.^103^ while performing experiments to determine the thermal resistance between solids and liquid helium II and has been later observed by Alexeev et al.^102^ in MD simulations involving the graphene–water interface.

Another parameter to describe the liquid structure at the interface is the density depletion length (DDL):
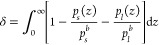
20where *p*_*s*_(*z*) and *p*_*l*_(*z*) are the solid and liquid densities, respectively, along the *z* axis where *z* = 0 is defined as the solid–liquid interface. *p*_*s*_^*b*^ and *p*_*s*_^*b*^ are the bulk solid and liquid densities, respectively. The DDL measures the influence of the solid into the liquid phase, indicating layering and density variation at the interface, and provides a quantification of the equilibrium distance between phases. Previous studies have used the DDL as a unifying parameter to reconcile plane-dependent ITR results across various Si and graphene-coated Si/water interfaces.^15,104^ A plot of *G*_*k*_ against δ is shown in Figure S12 in the Supporting Information. We observed that increasing δ resulted in an inversely proportional reduction in *G*_*k*_. A larger δ signifies less pronounced liquid layering at the interface and therefore a reduced coupling between the phases, leading to a lower ITC.

### Work of Adhesion

The dependence of the ITR on the solid–liquid interaction strength highlights the importance of carefully selecting an accurate potential to model these interactions. Due to the inherent challenges associated with the experimental determination of the ITR at solid–liquid interfaces,^2^ there is a lack of experimental data to use as a benchmark for these simulations. In the absence of such experimental data, we consider alternative methodologies to identify the optimal parameters for describing the solid–liquid interactions.

By comparing the simulation results to experimental data for the surface wettability of hematite by PAO4,^105^ it is possible to select which potential best describes solid–liquid interactions. Various different types of molecular dynamics simulations can be used to predict the wettability of solid surfaces. For example, the contact angle can be quantified through monitoring the geometry of nanodroplets or free energy-based methods.^74^ Alternatively, molecular dynamics simulations can be used to directly quantify the work of adhesion.^77^ We found the latter to be the most instructive for our systems. Previously, experimental values for the work of adhesion have been used to tune the solid–liquid LJ interaction strength for graphene–water systems.^72^

**Figure 6:**
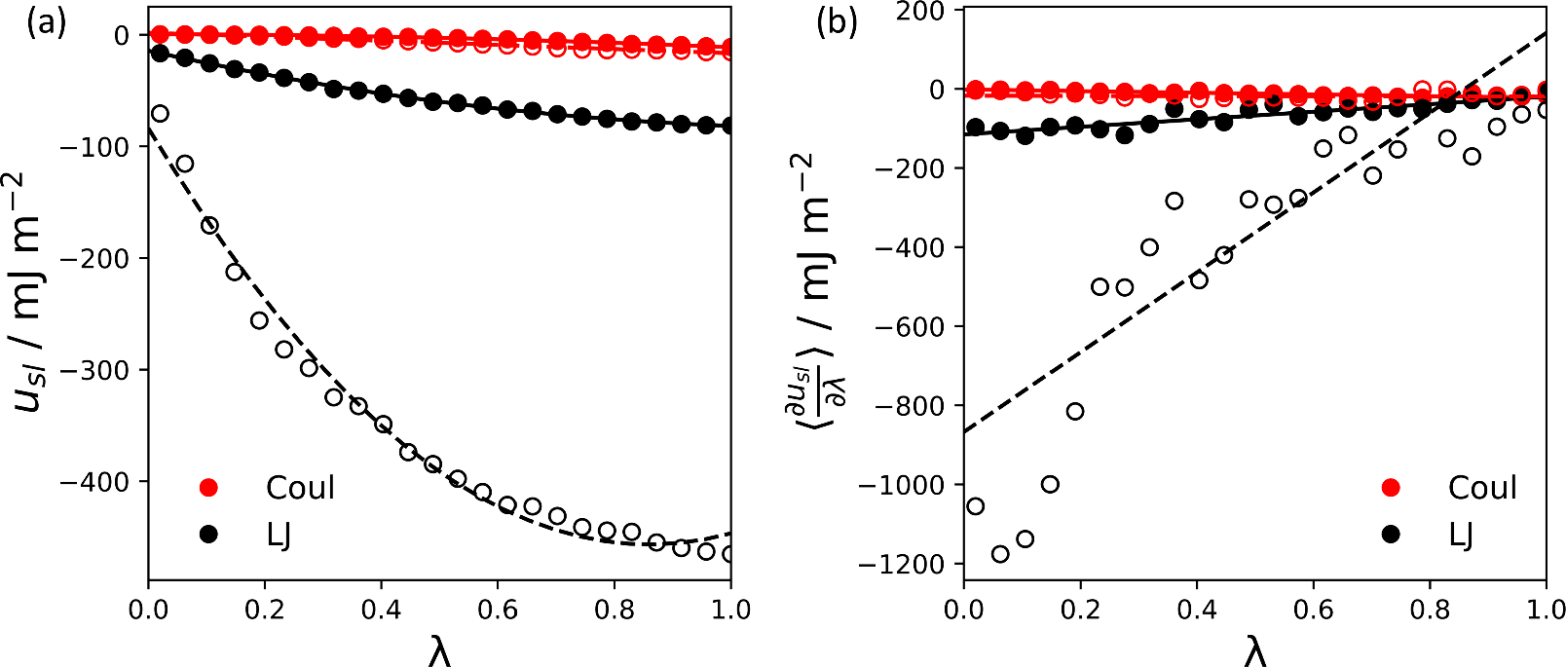
(a) Solid–liquid potential energy, *u*_*sl*_, plotted as a function of the scaling parameter, λ. Fitted lines show polynomial fits of *u*_*sl*_. (b) Corresponding derivative, , plotted as a function of λ. Numerical and analytical derivatives are represented by the markers and lines, respectively. Coulombic and LJ contributions are plotted separately in red and black, respectively. ClayFF^35,63^ results are represented by the filled markers and solid lines and Savio results are represented by the unfilled markers and dashed lines.

The solid–liquid work of adhesion was calculated for the α-Fe_2_O_3_ interface for each of the selected solid–liquid potentials. The solid–liquid potential energy, *u*_*sl*_, and corresponding derivative, , for the weakest (ClayFF^35,63^) and strongest (Savio et al.^34^) are shown as a function of λ in Figure 6. The Coulombic and LJ components of the solid–liquid potential energy, *u*_*sl*(*C*)_ and *u*_*sl*(*LJ*)_, respectively, are shown separately. We observe that, as λ is scaled toward zero, the contributions of both components tend toward zero. As the strength of the solid–liquid LJ potential was increased from ClayFF^35,63^ to Savio et al.,^34^ the LJ contribution to the work of adhesion, *W*_*sl*(*LJ*)_, increased significantly. The Coulombic contribution, *W*_*sl*(*C*)_, was much smaller and was essentially unchanged between both solid–liquid potentials. These observations were expected since the PAO4 fluid is nonpolar and the partial charges for both solid–liquid potentials were identical. The overall work of adhesion *W*_*sl*_ was calculated using eq 18.

**Figure 7:**
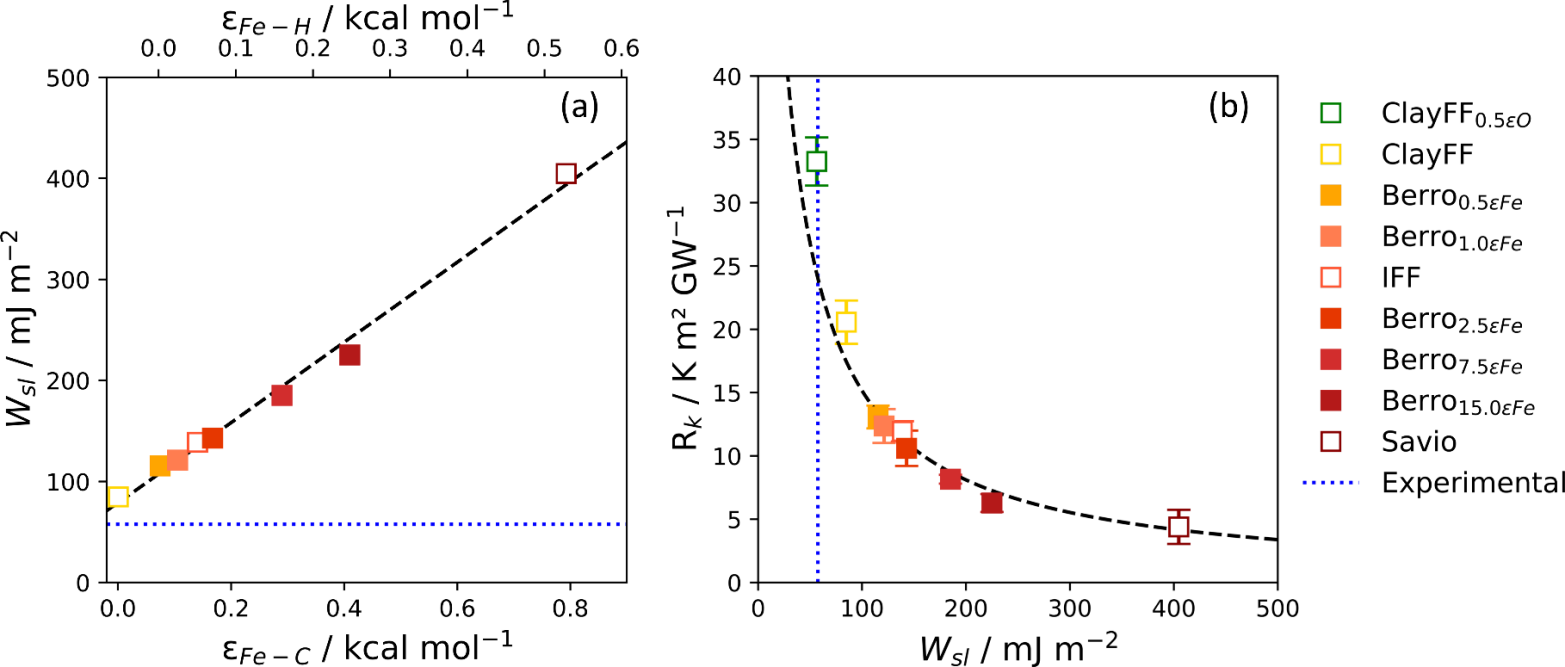
(a) Solid–liquid work of adhesion, *W*_*sl*_, plotted as a function of of the LJ well-depth parameter for iron carbon interactions, ε_Fe–C_ (lower axis), and iron–hydrogen interactions, ε_*Fe*–H_ (upper axis). The dashed black line is a linear fit. (b) ITR, *R*_*k*_, plotted as a function of *W*_*sl*_, dashed black line is a linear fit of *W*_*sl*_ versus *R*_*k*_^–1^. Filled markers represent potentials where only ε_Fe–PAO_ was changed. Unfilled markers represent potentials where ε_O–PAO_ parameter was also changed. Dotted blue lines show *W*_*sl*_ calculated from contact angle experiments by Kalin and Polajnar.^105^

The solid–liquid work of adhesion is shown in Table 4 for each of the eight potentials and plotted against the solid–liquid interaction strength, ε_*Fe*–C_ and ε_*Fe*–H_, in Figure 7 (a). We observed a linear increase of *W*_*sl*_ with ε_*Fe*–C_ and ε_*Fe*–H_. Since *W*_*sl*_ ∝(1 + cos θ),^15^ this result was in agreement with the scaling relationship, ε ∼ (1 + cos θ), proposed by Sendner et al.^106^ from molecular dynamics simulations of the water/diamond interface. This relationship has been further observed in molecular dynamics simulations for Si(111) and (100) surfaces in contact with water.^15^ The ITR is plotted as a function of *W*_*sl*_ in Figure 7 (b). We observed that *R*_*k*_ ∼ 1/*W*_*sl*_ and therefore *G*_*k*_ ∼ *W*_*sl*_, as shown in Figure S13 in the Supporting Information. This relationship has been established in a number of previous experimental and NEMD simulation studies.^15,71,95,107,108^

**Table 4:** Solid–Liquid Work of Adhesion, *W*_*sl*_, and Interfacial Thermal Resistance, *R*_*k*_, Calculated for Different Solid–Liquid LJ Potentials

Potential	*W*_*sl*_/mJ m^–2^	*R*_*k*_/K m^2^ GW^–1^
Experimental^105^	57.5	-
ClayFF_0.5 ε_*O*__	56.3 ± 0.1	33.3 ± 1.9
ClayFF	84.9 ± 1.4	20.6 ± 1.7
Berro_0.5 ε_*Fe*__	116.8 ± 1.5	13.1 ± 0.9
Berro_1.0 ε_*Fe*__	123.7 ± 2.5	12.4 ± 1.3
INTERFACE-FF	139.9 ± 1.2	11.9 ± 0.8
Berro_2.5 ε_*Fe*__	143.9 ± 0.9	10.6 ± 1.4
Berro_7.5 ε_*Fe*__	182.6 ± 2.6	8.2 ± 0.4
Berro_15.0 ε_*Fe*__	217.7 ± 7.4	6.3 ± 0.7
Savio	404.7 ± 5.9	4.4 ± 1.4

The dependence of the ITR on *W*_*sl*_ can be understood by considering the coupling of low frequency phonon modes across the interface. As solid–liquid interaction strength increases, the low frequency phonon modes of the solid and the liquid couple more effectively. This increased coupling allows for more efficient energy transfer across the interface, reducing ITR.^99,109^ This theory is supported by the reduction in the PAO4 DOS between 0 and 100 cm^–1^ and the Fe_2_O_3_ DOS peaks at 100 and 150 cm^–1^. The stronger coupling results in a redistribution in vibrational energy, lowering the DOS in the solid and liquid at specific frequency ranges.

We found that *W*_*sl*_ was overestimated for all of the solid–liquid potentials compared to the value determined from contact angle expriments by Kalin and Polajnar (57.5 mJ m^–2^).^105^ These results indicate that all of the solid–liquid potentials overestimate the interaction strength determined experimentally.

To address the overestimation of *W*_*sl*_, we introduced a new solid–liquid potential, ClayFF_0.5 ε_*O*__, which halved the value of ε_*O*_ in ClayFF^35,63^ for the solid–liquid LJ interactions (ε_*O*_ = 0.0777 kcal mol^–1^), while leaving the remaining parameters unchanged. We decided to change the ε_*O*_ parameter because the ε_*Fe*_ parameter of ClayFF was already so small (ε_*Fe*_ = 9.0 × 10^–6^) that reducing it further would likely have a negligible impact on the *W*_*sl*_. We then recalculated *W*_*sl*_ with this new potential. The results of these simulations are detailed in Table 4. This adjustment yielded a work of adhesion (*W*_*sl*_ = 56.3 mJ m^–2^) that closely matched the value determined from experiments.^105^

To achieve this agreement with *W*_*sl*_ from contact angle experiments,^105^ we reduced the strength of the O_*surf*_-C_*fluid*_ and O_*surf*_-H_*fluid*_ LJ interactions, which have been carefully parametrized (at least for O_*fluid*_-C_*fluid*_ and O_*fluid*_-H_*fluid*_) in the molecular force field.^40,41^ This raises the question as to whether our system is fully representative of the state of solid–liquid interface in the contact angle experiments. Experimental studies have shown that surface hydration and hydroxylation can occur on hematite surfaces at relative humidities as low as 0.1%.^110^ The relative humidity was not reported for the contact angle experiments used to calculate the work of adhesion.^105^ However, since no controlled atmosphere was used, it is very likely that an interfacial hydroxyl and/or water layer was present between the hematite and the PAO4 in the contact angle experiments. The presence of these layers could screen the interactions between the Fe_2_O_3_ and PAO4, reducing adhesive forces with respect to dry interfaces. Moreover, surface contaminants and surface roughness are not accounted for in this model. These real-world factors could influence the wettability and ITR and incorporating them into future NEMD simulations may be essential to accurately represent system behavior. This highlights the need for further investigation into this area in future studies.

## Conclusions

In this study, we investigated the ability of a wide range of classical force fields to model the thermal properties of iron oxide solid (hematite) and a hydrocarbon liquid (PAO4). We benchmarked the ability of the models to predict the experimental density and thermal conductivity, as well as viscosity for the liquid. We observed significant deviations from experimental values when testing many of the force fields, demonstrating the importance of careful selection when simulating such systems.

For the PAO4 fluid, all of the force fields were able to accurately predict the density. The three all-atom force fields overestimated the thermal conductivity by a similar amount (∼50%), while the united-atom force field under predicted the thermal conductivity by 27%. From viscosity calculations, we saw significant under predictions at high and low temperatures with CHARMM-36 and TraPPE-UA, and over predictions using AMBER. On the other hand, LOPLS-AA was found to be in good agreement with experimental data and was therefore chosen as the force field that best captures the behavior of PAO4.

For the hematite surface, we tested four different force fields and again observed that, while all force fields could accurately predict the density, the predicted thermal conductivity varied significantly from experimental results. The most accurate force field we tested was the Morse potential. The thermal conductivity calculated using this model was in excellent agreement with experimental data obtained in all three crystallographic directions. ClayFF and INTERFACE-FF were found to under predict the thermal conductivity by 71% and 47% respectively, while the harmonic model significantly overpredicted it by >500%. These large discrepancies in solid thermal conductivity are shown not to be important to the measurement of ITR in subsequent NEMD simulations.

Using the Morse potential to model the hematite surface and LOPLS-AA for the PAO4 fluid, we calculated the ITR of the hematite/PAO4 interface for a range of solid–liquid interaction potentials (4–21 K m^2^ GW^–1^). Based on our calculations, the ITR will be relevant in length scales corresponding to Kapitza lengths *l*_*K*_ = *κ G*_*K*_ ∼ 1–10^2^ nm. The potentials tested in this work varied in the strength of the Fe–C and Fe–H LJ interactions. We observed a strong dependency of the ITR on the strength of Fe–C and Fe–H interactions, with a stronger potential resulting in a lower thermal conductivity. To rationalize this behavior we calculated the VDOS for the hematite and PAO4 regions adjacent to the interface as well as the overlap integral of the two spectra. We observed no significant changes in the vibrational spectra for the hematite or PAO4 regions or the overlap integral as the strength of the interactions were increased. However, we observed that a significant increase in the first adsorption layer density of the fluid as the strength of the solid–liquid interactions was increased. We therefore conclude that the decrease in ITR arises, not from changes in the vibrational spectra of the two components, but from the increased density of PAO4 molecules close to the surface, and therefore increased availability of energy carriers, as the solid–liquid interaction strength was increased.

Since the ITR has not been experimentally measured for the hematite/PAO4 interface, we performed a series of work of adhesion calculations using the different solid–liquid potentials. We observed that, for all of the solid–liquid potentials tested, the work of adhesion was over predicted compared to the value derived from contact angle experiments. To reconcile our simulation results with the experiments, we had to reduce the strength of the O_*surf*_–C_*fluid*_ and O_*surf*_–H_*fluid*_ interactions. This lead to a much larger ITR (33 K m^2^ GW^–1^) than for the other potentials.

These findings indicate that a more detailed representation of the solid–liquid interface might be needed to accurately reproduce experimental properties. We propose that the inclusion of a hydroxyl and/or water layer at between hematite and PAO4 may provide a more accurate model of the interface in the experiments. Further investigation in future studies is necessary to increase our understanding and improve the accuracy of molecular simulations of the hematite/PAO4 interface. The methodology proposed here could also be readily extended to study immersion cooling fluids for other applications with different interfaces, such as CPUs, data centers, and photovoltaics.

## Supporting Information Available

Further details on viscosity calculation methods, parameters used for interfacial thermal resistance calculations and additional supporting results to those presented here (PDF)
