# Supplementary material for: Molecular Simulations of Thermal Transport across Iron Oxide–Hydrocarbon Interfaces
Source: ACS Appl Mater Interfaces. 2024 Oct 15;16(43):59452–67. doi: 10.1021/acsami.4c09434 (PMC11533160; doi:10.1021/acsami.4c09434)
Supplement: Supplementary file 1 — am4c09434_si_001.pdf [file am4c09434_si_001.pdf]

Supporting Information: Molecular Simulations of Thermal Transport across Iron Oxide-Hydrocarbon Interfaces

Fionn Carman,[*,†] James P. Ewen,[*,†] Fernando Bresme,[‡] Billy Wu,[¶] and Daniele Dini[†]

†*Department of Mechanical Engineering, Imperial College London, London SW7 2AZ, UK*

‡*Department of Chemistry, Molecular Sciences Research Hub, Imperial College London, London W12 0BZ, UK*

¶*Dyson School of Design Engineering, Imperial College London, London SW7 2AZ, UK*

E-mail: fionn.carman18@imperial.ac.uk; j.ewen@imperial.ac.uk

Liquid Thermal Conductivity

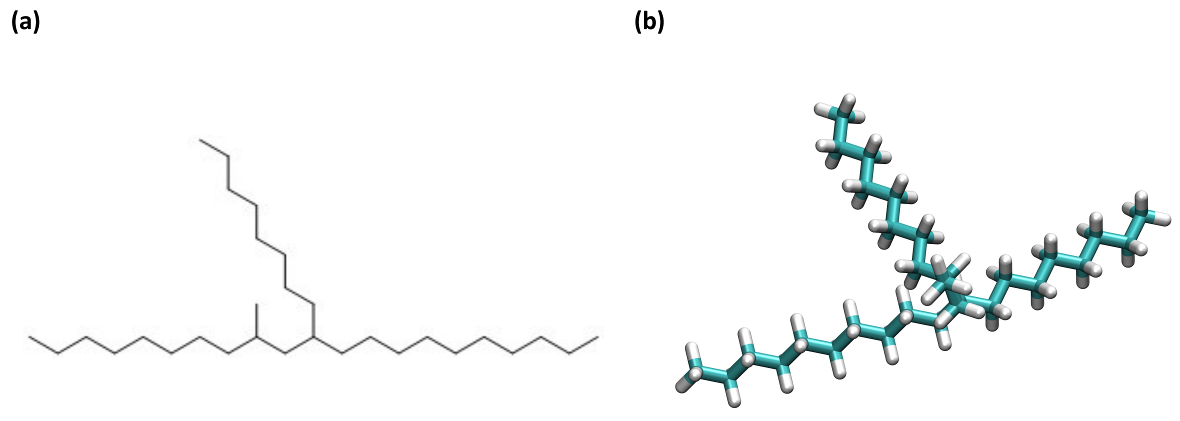

Figure S1: (a) Skeletal formula of polyalphaolefin (PAO4). (b) Three-dimensional representation of the PAO4 molecule as visualized in VMD software (cyan: carbon, white: hydrogen).

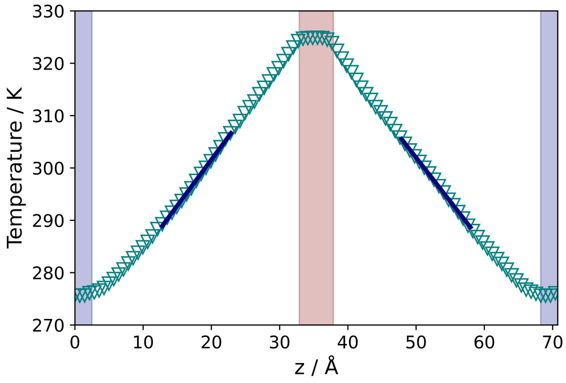

Figure S2: A typical time averaged temperature profile of PAO4 during NEMD simulations. Hot and cold thermostatted regions are shaded in red and blue respectively and the navy lines show the linear fitted region.

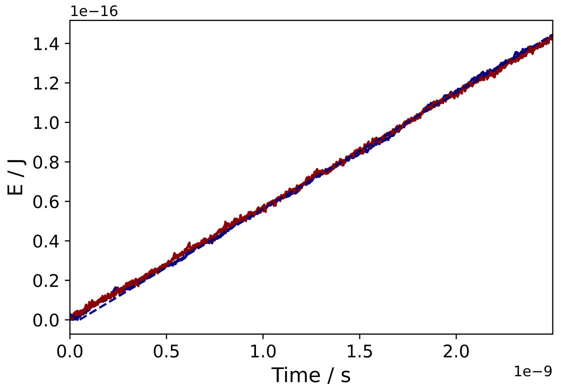

Figure S3: Energy exchanged in the hot and cold thermostats, shown by the red and blue lines respectively. The sign of the energy exchanged in the hot thermostat has been flipped to enable comparison of the two data sets.

Liquid Viscosity

To ensure we obtained accurate results in our simulations, we conducted preliminary tests to determine the appropriate autocorrelation time period, d. The autocorrelation time period represents the time scale over which the PACF was sampled and was systematically varied between 5 and 2500 ps by increasing the number of autocorrelation terms, p, whilst keeping a constant sample rate $s = 10$ fs. Figure S4 (a) shows the viscosity profiles for each correlation time, calculated by integrating the PACF, Equation 6. The viscosity was calculated by taking the mean of the viscosity profile once it had converged. Figure S4 (b) shows the viscosity plotted against the correlation time. We observe that increasing the autocorrelation time period results in an increase in viscosity estimates, before converging for time periods greater than 1000 ps, indicating that the time period was sufficient for the PACF to decay to zero. Notably, it can be seen in Figure S4 (a) that increasing the autocorrelation time period increases the time taken for the viscosity profile to converge. Therefore longer production runs are required for longer autocorrelation time periods. We chose to use a correlation time of 1000 ps in all future simulations to obtain accurate estimates for the viscosity whilst also minimising the computational cost.

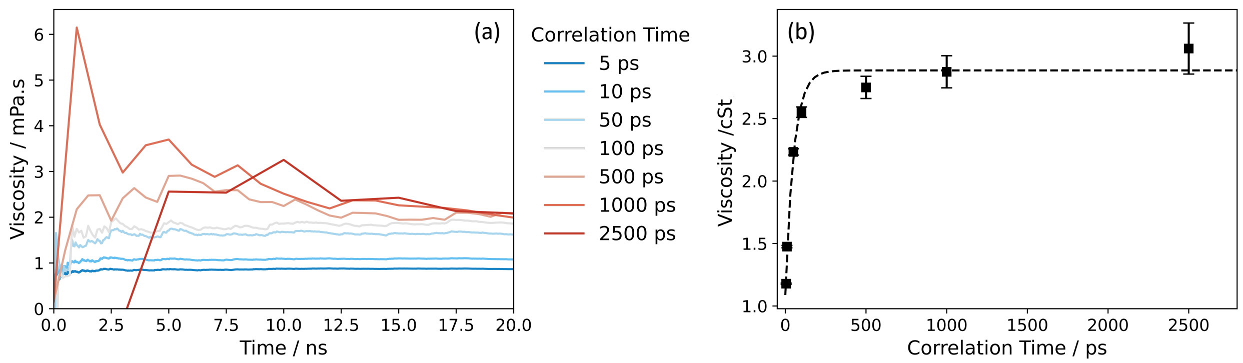

Figure S4: (a) Viscosity profiles for different correlation times. (b) Mean viscosity as a function of correlation time.

Solid Thermal Conductivity

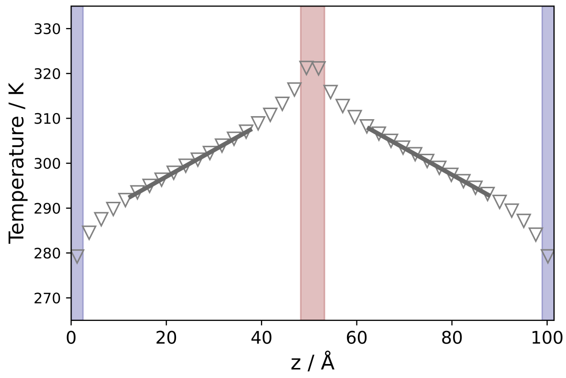

Figure S5: Typical temperature profile for Fe_2O_3 surfaces during NEMD simulations. Hot and cold thermostatted regions are shaded in red and blue respectively and the dark grey lines show the linear fitted region.

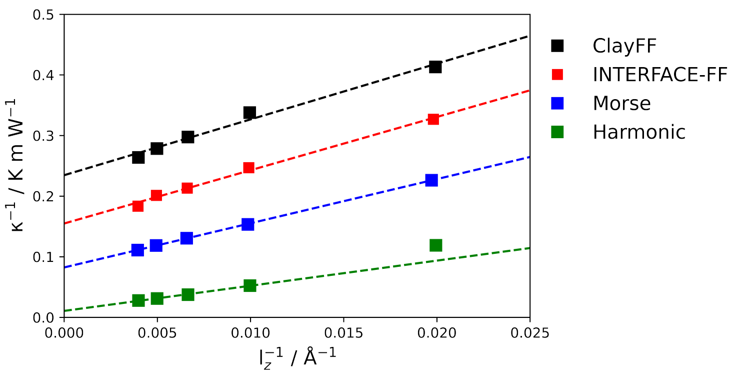

Figure S6: The inverse of the thermal conductivity ($1/\lambda$), calculated at 300 K and 1 atm in the [100] direction, plotted against the inverse of the lateral box length ($1/l_z$) for the 4 different force fields.

Interfacial Thermal Resistance

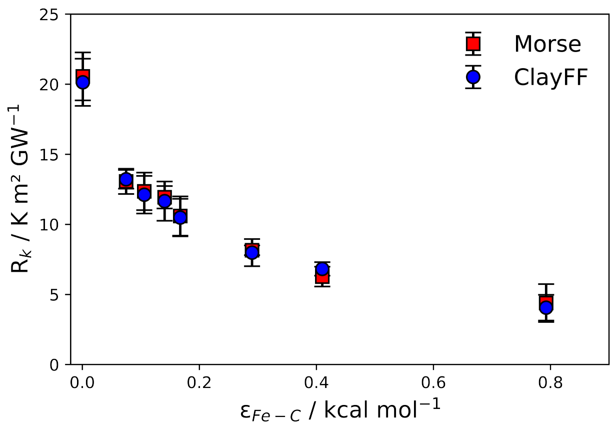

Figure S7: A comparison of ITR calculated using partial charges and LJ parameters from the Morse potential[6] (red) and ClayFF[1,2] (blue) for solid-solid interactions.

Table S1: LJ parameters used for the different solid-liquid interaction potentials.

Potential	ε / kcal mol^{-1}					
	Fe-C	Fe-H (CH_3)	Fe-H (CH_2)	O-C	O-H (CH_3)	O-H (CH_2)
$\text{ClayFF}_{0.5\varepsilon_O}$ [1,2]	0.000771	0.000520	0.000486	0.0716	0.0483	0.0452
ClayFF [1,2]	0.000771	0.000520	0.000486	0.101	0.0683	0.0639
$\text{Berro}_{0.5\varepsilon_{Fe}}$ [3]	0.0749	0.0505	0.0473	0.106	0.0714	0.0668
$\text{Berro}_{1.0\varepsilon_{Fe}}$ [3]	0.106	0.0714	0.0668	0.106	0.0714	0.0668
INTERFACE-FF [4]	0.141	0.0949	0.0888	0.115	0.0775	0.0725
$\text{Berro}_{2.5\varepsilon_{Fe}}$ [3]	0.167	0.113	0.106	0.106	0.0714	0.0668
$\text{Berro}_{7.5\varepsilon_{Fe}}$ [3]	0.290	0.196	0.183	0.106	0.0714	0.0668
$\text{Berro}_{15.0\varepsilon_{Fe}}$ [3]	0.410	0.277	0.259	0.106	0.0714	0.0668
Savio [5]	0.793	0.534	0.500	0.118	0.0794	0.0743

Potential	σ / Å					
	Fe-C	Fe-H (CH_3)	Fe-H (CH_2)	O-C	O-H (CH_3)	O-H (CH_2)
$\text{ClayFF}_{0.5\varepsilon_O}$ [1,2]	3.78	3.19	3.19	3.33	2.81	2.81
ClayFF [1,2]	3.78	3.19	3.19	3.33	2.81	2.81
$\text{Berro}_{0.5\varepsilon_{Fe}}$ [3]	2.77	2.35	2.35	3.22	2.72	2.72
$\text{Berro}_{1.0\varepsilon_{Fe}}$ [3]	2.77	2.35	2.35	3.22	2.72	2.72
INTERFACE-FF [4]	2.54	2.15	2.15	3.31	2.80	2.80
$\text{Berro}_{2.5\varepsilon_{Fe}}$ [3]	2.77	2.35	2.35	3.22	2.72	2.72
$\text{Berro}_{7.5\varepsilon_{Fe}}$ [3]	2.77	2.35	2.35	3.22	2.72	2.72
$\text{Berro}_{15.0\varepsilon_{Fe}}$ [3]	2.77	2.35	2.35	3.22	2.72	2.72
Savio [5]	2.85	2.41	2.41	3.22	2.72	2.72

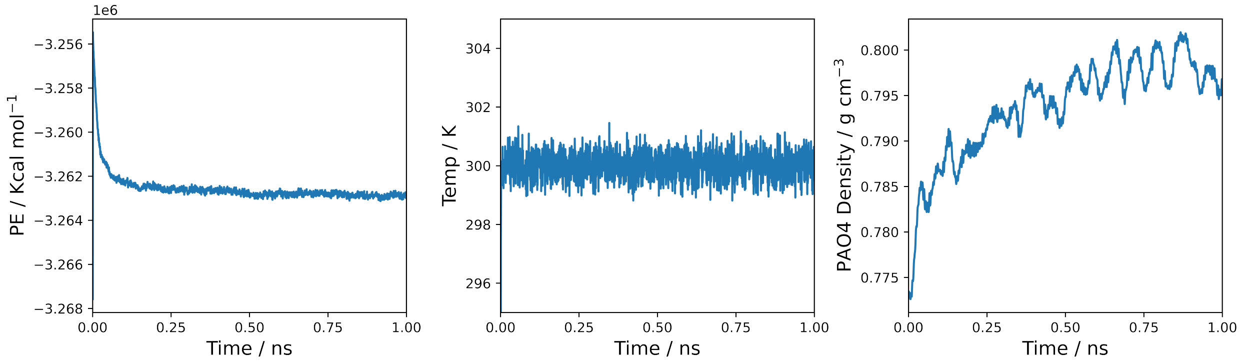

Figure S8: Potential energy, temperature and density in the liquid region displayed as a function of energy during the NPT equilibration stage, shown for the IFF interfacial potential.

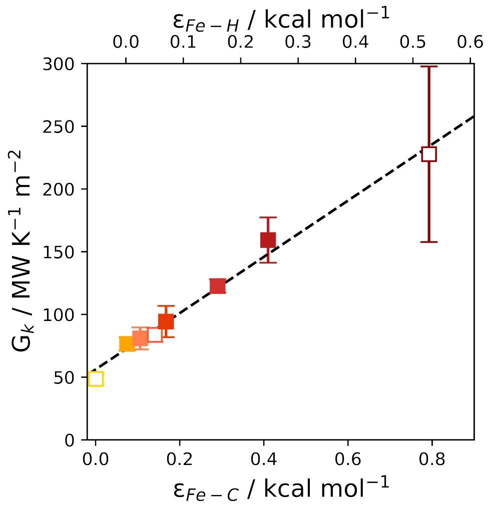

Figure S9: Interfacial thermal conductance, (G_k), plotted as a function of ε_{Fe-C} (lower axis) and ε_{Fe-H} (upper axis).

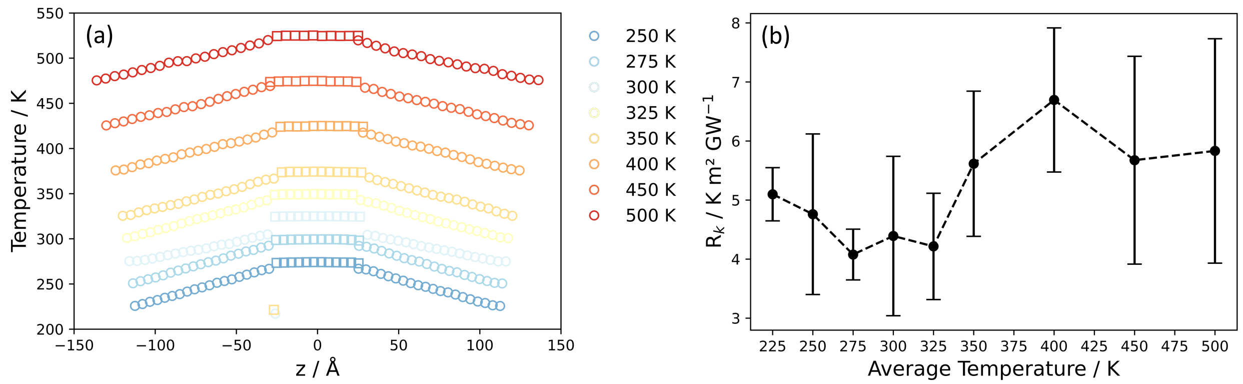

Figure S10: (a) Temperature profiles shown for different temperatures (b) ITR plotted as a function of average temperature.

Vibrational Density of States

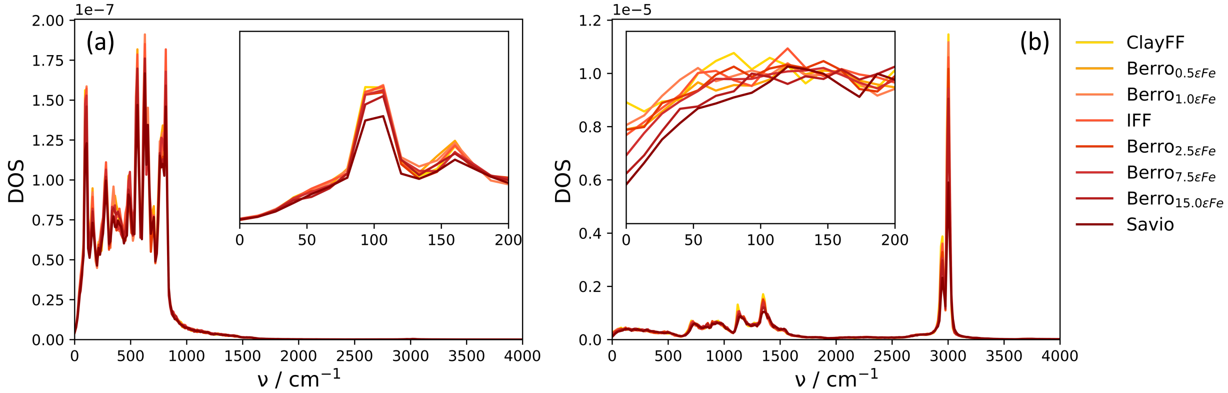

Figure S11: VDOS calculated for PAO4 (a) and Fe_2O_3 (b) for the different solid-liquid interaction potentials. The low frequency, transverse modes are expanded in the insets.

Interfacial Liquid Structure

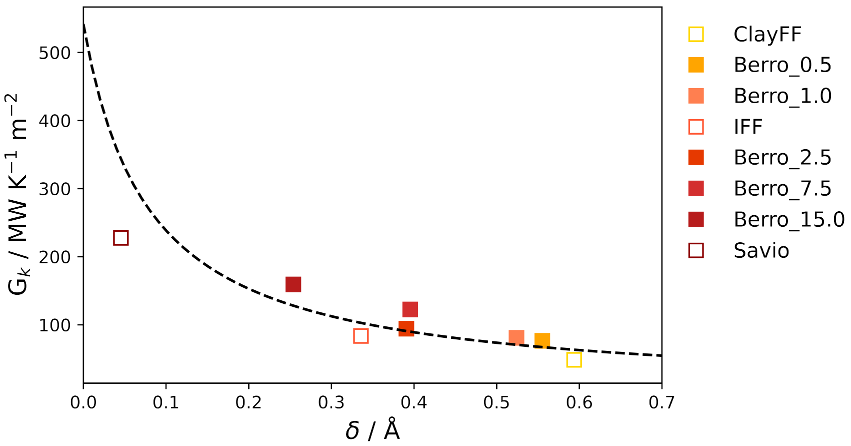

Figure S12: Interfacial thermal conductance, (G_k), plotted as a function of the density depletion length, δ.

Work of Adhesion

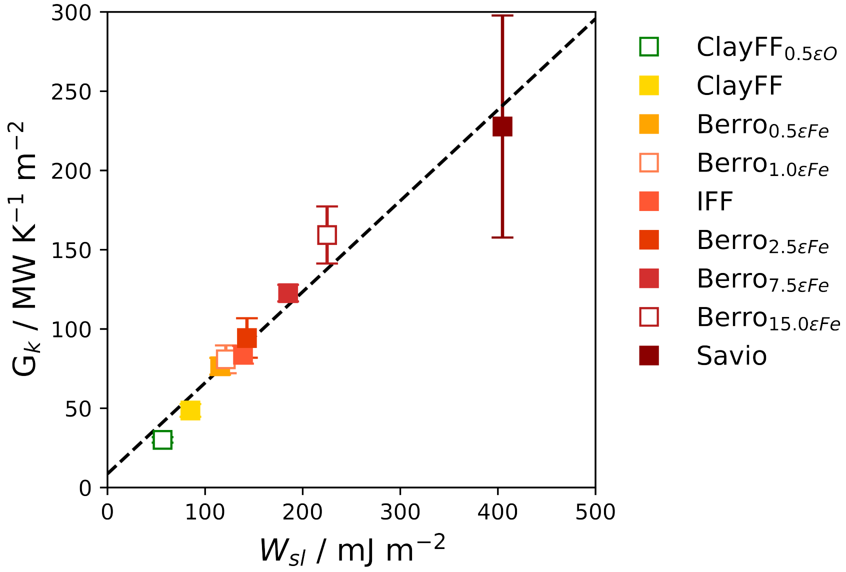

Figure S13: Interfacial thermal conductance, (G_k), plotted as a function of solid-liquid work of adhesion, (W_{sl}).

References

(1) Cygan, R. T.; Liang, J.-J.; Kalinichev, A. G. Molecular Models of Hydroxide, Oxyhydroxide, and Clay Phases and the Development of a General Force Field. *The Journal of Physical Chemistry B* **2004**, *108*, 1255–1266.

(2) Kerisit, S. Water structure at hematite-water interfaces. *Geochimica et Cosmochimica Acta* **2011**, *75*, 2043–2061.

(3) Berro, H.; Fillot, N.; Vergne, P. Molecular dynamics simulation of surface energy and ZDDP effects on friction in nano-scale lubricated contacts. *Tribology International* **2010**, *43*, 1811–1822.

(4) Kanhaiya, K.; Nathanson, M.; in 't Veld, P. J.; Zhu, C.; Nikiforov, I.; Tadmor, E. B.; Choi, Y. K.; Im, W.; Mishra, R. K.; Heinz, H. Accurate Force Fields for Atomistic Simulations of Oxides, Hydroxides, and Organic Hybrid Materials up to the Micrometer Scale. *Journal of Chemical Theory and Computation* **2023**, *19*, 8293–8322.

(5) Savio, D.; Fillot, N.; Vergne, P.; Zaccheddu, M. A Model for Wall Slip Prediction of Confined n-Alkanes: Effect of Wall-Fluid Interaction Versus Fluid Resistance. *Tribology Letters* **2012**, *46*, 11–22.

(6) Severin, J.; Jund, P. Thermal conductivity calculation in anisotropic crystals by molecular dynamics: Application to α-Fe2O3. *The Journal of Chemical Physics* **2017**, *146*, 054505.
